# Co-Inoculation of *Bacillus* *velezensis* Strain S141 and *Bradyrhizobium* Strains Promotes Nodule Growth and Nitrogen Fixation

**DOI:** 10.3390/microorganisms8050678

**Published:** 2020-05-07

**Authors:** Surachat Sibponkrung, Takahiko Kondo, Kosei Tanaka, Panlada Tittabutr, Nantakorn Boonkerd, Ken-ichi Yoshida, Neung Teaumroong

**Affiliations:** 1 School of Biotechnology, Institute of Agricultural Technology, Suranaree University of Technology, Nakhon Ratchasima 30000, Thailand; surachat1985@hotmail.com (S.S.); panlada@sut.ac.th (P.T.); nantakon@sut.ac.th (N.B.); 2 Graduate School of Science, Technology and Innovation, Kobe University, Kobe 657-8501, Japan; kondo.t.7.4@gmail.com; 3 Organization of Advanced Science and Technology, Kobe University, Kobe 657-8501, Japan; ktanaka@people.kobe-u.ac.jp

**Keywords:** PGPR, *Bradyrhizobium*, Co-inoculation, Soybean, Nodulation

## Abstract

The objective of this research was to evaluate the PGPR effect on nodulation and nitrogen-fixing efficiency of soybean (*Glycine* max (L.) Merr.) by co-inoculation with *Bradyrhizobium* *diazoefficiens* USDA110. Co-inoculation of *Bacillus* *velezensis* S141 with USDA110 into soybean resulted in enhanced nodulation and N2-fixing efficiency by producing larger nodules. To understand the role of S141 on soybean and USDA110 symbiosis, putative genes related to IAA biosynthesis were disrupted, suggesting that co-inoculation of USDA110 with S141Δ*yhcX* reduces the number of large size nodules. It was revealed that *yhcX* may play a major role in IAA biosynthesis in S141 as well as provide a major impact on soybean growth promotion. The disruption of genes related to cytokinin biosynthesis and co-inoculation of USDA110 with S141Δ*IPI* reduced the number of very large size nodules, and it appears that IPI might play an important role in nodule size of soybean–*Bradyrhizobium* symbiosis. However, it was possible that not only IAA and cytokinin but also some other substances secreted from S141 facilitate *Bradyrhizobium* to trigger bigger nodule formation, resulting in enhanced N2-fixation. Therefore, the ability of S141 with Bradyrhizobium co-inoculation to enhance soybean N2-fixation strategy could be further developed for supreme soybean inoculants.

## 1. Introduction

The nitrogen-fixing symbiosis between leguminous plants and rhizobia is capable of reducing the need for nitrogen fertilizer. Nevertheless, many factors obstruct the nodulation of leguminous plant roots including biotic and abiotic factors that suppress the nodule formation. The lack of nodule formation of leguminous plant roots may result from the incompatibility of the host–microbe symbiont as well as the function of biological molecules such as hormones, polysaccharides, and flavonoids [1]. Bradyrhizobium can form either general or specific symbioses. This suggests that several legume species may be able to nodulate only one strain of Bradyrhizobium, while other Bradyrhizobium species may be able to nodulate only one legume species [2]. Among rhizobia, *Bradyrhizobium diazoefficiens* USDA110 is the most agriculturally important species because it has the ability to form root nodules on soybean (*Glycine max* (L.) Merr.) [3]. A positive relationship between the sizes of soybean nodules and nitrogen fixation ability was shown by Weisz and Sinclair [4] who reported that larger nodules (ca. 4 mm in diameter) had higher nitrogen-fixing activity than smaller ones (ca. 2 mm).

Plant growth-promoting rhizobacteria (PGPR) are commonly occurring soil bacteria that colonize plant roots and have benefited with plants through promoting plant growth [5]. Some PGPR strains improve nodulation, nitrogen fixation, and legume growth when co-inoculated with rhizobia. Examples of PGPR include *Azotobacter* [6,7], *Azospirillum* [8,9], *Bacillus* [10,11,12], *Pseudomonas* [13,14], *Serratia* [14,15], *Staphylococcus* [10], and *Streptomyces* [16,17], which all share a portion of common microhabitats in the root–soil interface. During the processes of root colonization, rhizobia and PGPR must interact [18]. The influence of *Rhizobium* and PGPR co-inoculation has been discovered in various symbiotic and plant growth parameters. Co-inoculation of *Rhizobium* spp. and *Azospirillum* spp. can improve the number of nodules by an increased number of root hairs and amount of flavonoids secreted from the roots when compared with *Rhizobium* single inoculation [9,19,20,21]. In addition, PGPR co-inoculation with either *B. diazoefficiens* USDA110 or *B. diazoefficiens* CB1809 produces high nodule number, increased nodule dry weight, and increased seed yield more than single inoculation [8]. Moreover, co-inoculation with *B. diazoefficiens* USDA110 and *Bacillus subtilis* or *Staphylococcus* sp. increases number of active nodules and plant yield [10]. The probability that metabolites such as flavonoids and siderophores that stimulate the expression of nod gene might improve nodule formation has been proposed [22]. The advantageous effect on *Rhizobium*–legumes nodulation by PGPR has been differentially applied to their ability to synthesize phytohormones [23]. 

However, these hypotheses have not been completely confirmed. The rhizobacterium may not be effective and predominant in all environments. Mixtures of compatible rhizobacteria might have more benefits than single rhizobacterium inoculation in plant growth promotion. The co-inoculation of rhizobia and PGPR may show many effects on nodule morphology and legume growth. This may depend on the stage of the process modified via PGPR, but the effect of biological molecules produced via the co-inoculation process on soybean–bradyrhizobial symbiosis needs to be analyzed in more detail. Bacterial S141 strain is a PGPR isolated from soybean rhizosphere soil in Thailand by Prakamhang et al. [10]. S141 is capable of enhancing soybean growth and yields via co-inoculation with *B. diazoefficiens* strains USDA110 and THA6 in field experiments. The whole-genome sequence of S141 was identified to comprise a 3.97-Mb-long circular chromosome lacking a plasmid. The genome sequence of S141 encodes at least 3817 protein-coding genes. Based on 16S rRNA gene analysis and average nucleotide identity (ANI), the results indicate that strain S141 was classified as *B. velezensis*. Moreover, multiple genes that are functionally related to auxin and cytokinin biosynthesis were found in S141, and may play an important role in its ability to promote plant growth [24]. The overall N_2_-fixing efficacy and efficiency of the soybean–bradyrhizobial symbiosis could be enhanced by co-inoculation using cells or supernatant of S141 strain. This study aimed to examine the roles of some determinants derived from S141 that affect *Bradyrhizobium* co-inoculation that can enhance symbiosis, resulting in better nodulation, nitrogen fixation, nodule morphology, growth, and yield in soybean plants.

## 2. Materials and Methods 

### 2.1. Bacterial Strains and Growth Conditions

The bacterial strains used in this study are listed in Table 1. *Bradyrhizobium diazoefficiens* USDA110 and *B. diazoefficiens* CB1809 were cultured in yeast extract mannitol (YEM) [2] at 28 °C for 6 days to obtain bacterial cell numbers of 10^6^–10^8^ CFU mL^−1^. *Bacillus velezensis* strain S141 was grown in Luria–Bertani (LB) medium broth for 48 h at 30 °C to obtain bacterial cell numbers of 10^6^–10^8^ CFU mL^−1^. When necessary, the medium was supplemented with antibiotics: 5 μg mL^−1^ chloramphenicol, 1 μg mL^−1^ erythromycin, 10 μg mL^−1^ kanamycin, 8 μg mL^−1^ phleomycin, 100 μg mL^−1^ spectinomycin, and 100 μg mL^−1^ streptomycin. The early stationary phase of bacterial cultures was centrifuged at 4000 rpm for 10 min and washed with sterilized 0.85% (w/v) NaCl to take out the excess media and the cell pellet was resuspended in 0.85% (w/v) NaCl solution. Cultures were adjusted with 0.85% (w/v) NaCl solution to a final concentration of 10^6^ CFU mL^−^^1^ before inoculation into plants. 

### 2.2. Co-Inoculation Effect of S141 on Soybean–Bradyrhizobium Symbiosis 

The soybean (*Glycine max* (L.) Merr.) cultivars tested in these experiments were soybean cultivar Chiang Mai 60 (CM60), a recommended line for application in Thai field conditions, and soybean cultivar Enrei, a reference for Japanese domestic soybean cultivars. Seeds of soybean were grown in growth chambers by Leonard’s jar assemblies modification [27]. Soybean seeds were surface sterilized by full immersion in 70% ethanol for 30 s, followed by rinsing five times with sterilized water [28]. Seeds were then sown in sterilized grade-2 vermiculite in modified Leonard’s jars until seedling emergence (3 days) [29]. The Leonard’s jar experiment was conducted to estimate the co-inoculation effects of S141 with *B. diazoefficiens* USDA110 on soybean. The seedlings were inoculated separately with 1 mL of 10^6^ CFU mL^−1^ of S141 or USDA110 for the single inoculation and mixed in a ratio of 1:1 for co-inoculation treatment. The cell suspensions were replaced by sterilized distilled water in the control treatment. Plant growth conditions in the laboratory were controlled using a 16-h-day/8-h-night cycle at 28 °C/23 °C. The plants were watered regularly with N-free nutrient solution during the experiment [30]. The experiments were set up with 6–10 replicates for each treatment. The plants were grown for 45 days after inoculation (DAI). Nitrogenase activity was measured using the Acetylene Reduction Assay (ARA) [2]. Nodule number, nodule dry weight, shoot dry weight, root dry weight, and total plant dry weight were recorded. The tissues were placed in a hot air oven at 65 °C for 4 days before weighing (modified from [2]).

### 2.3. Co-Inoculation Effect of S141 and Biological Molecules Secreted from S141 on Soybean–Bradyrhizobium Symbiosis

S141 cell preparation was carried out as described in Section 2.1. The S141 supernatant (SN) was filtered through a 0.22 μm polytetrafluoroethylene (PTFE) membrane. The culture filtrate was used for single inoculation and co-inoculation treatments or kept at −80 °C for further experimentation. Leonard’s jar experiments was conducted as described in Section 2.2. The experiments were carried out to evaluate the co-inoculation effects of *B. diazoefficiens* USDA110 with cells or supernatant of S141 on soybean. The seedlings were inoculated separately with 1 mL of 10^6^ CFU mL^−1^ of S141, *B. diazoefficiens* USDA110, or CB1809 or 1 mL of the supernatant of S141 for the single inoculation and mixed in a ratio of 1:1 for co-inoculation treatment. The cell suspension was replaced by sterilized distilled water in the control treatment. Plant growth conditions in the laboratory were followed as described in Section 2.2. 

### 2.4. Effect of Bradyrhizobium Co-Inoculation Dose with Biological Molecules Secreted from S141 on Soybean–Bradyrhizobium Symbiosis

Soybean cultivar CM60 seedlings co-inoculated with cells or supernatant of S141 at 10^6^ cells seed^−1^ were mixed in a ratio of 1:1 with USDA110 at six inoculum doses: 10^3^, 10^4^, 10^5^, 10^6^, 10^7^, and 10^8^ CFU mL^−1^. The USDA110 cells or supernatant of S141 culture (1 mL seed^−1^) were used for single inoculation. The cell suspension was replaced by sterilized distilled water in the control treatment. Plants were grown in growth chambers using modified Leonard’s jar assemblies and sampled at 45 DAI. The nodule number, nodule dry weight, shoot dry weight, root dry weights, and nitrogenase activity using ARA was measured [2].

### 2.5. Effect of Co-Inoculation with S141 on Bradyrhizobium Competition

Seeds of soybean cultivar Chiang Mai 60 were cultivated in a pot. Seeds were surface sterilized and germinated on wet tissue paper [2]. Bradyrhizobial strain GUS-tagged USDA110 [8] was cultured in yeast extract mannitol (YEM) medium broth supplemented with antibiotics: 100 μg mL^−1^ streptomycin and 100 μg mL^−1^ spectinomycin at 28 °C for 6 days to obtain bacterial cell numbers of 10^6^–10^8^ CFU mL^−1^. The pot experiments used local soil collected from fields based on cropping history and were conducted to evaluate the co-inoculation effects of S141 with GUS-tagged USDA110 on soybean growth. The chemical characteristics of the soil are listed in Table S1. The seedlings were inoculated separately with 1 mL of 10^6^ CFU mL^−1^ of S141 or GUS-tagged USDA110 for the single inoculation and mixed in a ratio of 1:1 for co-inoculation. The cell suspension was replaced by sterilized distilled water in the control treatment. Plants were grown in greenhouse conditions. The plants were watered regularly with tap water during the experiment. There were five replicates for each treatment. Plants were harvested at 45 DAI, the nodule formation of the GUS-tagged USDA110 strain was checked on soybean hosts by the GUS-staining method. Histochemical staining of plant material was performed as described by Jefferson [31]. The explants were subjected to vacuum infiltration for 10 min before incubating them overnight at 37 °C. The nodule number, root dry weight, and shoot dry weight were recorded.

### 2.6. Colonization of S141 on Soybean–Bradyrhizobium Symbiosis

The green fluorescent protein (GFP) and phleomycin resistance gene were activated in S141 by insertion to *tuf* promoter gene. The DNA fragments including upstream and downstream regions of *tuf* promoter were amplified by PCR with S141 chromosomal DNA as a template using primers tuf-uF/tuf-uR in normal condition for the upstream fragment and tuf-dF/tuf-dR for the downstream fragment (Table S2). The additional DNA fragment of *B. subtilis* strain TSU077 (Table 1) containing the phleomycin resistance gene was amplified using primers phl-F/phl-R (Table S2), and strain 168:ytsJ:gfp (Table 1) containing the green fluorescent protein was amplified using primers gfp-F/gfp-R (Table S2). The recombinant PCR was applied to ligate four DNA fragments using primers tuf-uF/tuf-dR to sandwich the green fluorescent protein and phleomycin resistance gene between the upstream and downstream regions of *tuf* promoter. The recombinant PCR fragment was transformed into S141 by natural competent method conferring phleomycin resistance and yielding the new strain, S141::GFP (*tuf::gfp::phle^r^*), which was used as the GFP-tagged strain for the colonization on soybean–*Bradyrhizobium* symbiosis in this study. The Leonard’s jar experiment (Section 2.2) was conducted to reveal the colonization of S141 on soybean–*Bradyrhizobium* symbiosis. S141::GFP was grown in Luria–Bertani (LB) medium broth supplemented with phleomycin 8 μg mL^−1^ at 30 °C for 48 h. The seedlings were inoculated separately with 1 mL of 10^6^ CFU mL^−1^ of S141::GFP or USDA110 for the single inoculation and mixed in a ratio of 1:1 for co-inoculation treatment. The cell suspension was replaced by sterilized distilled water in the control treatment. Plant growth conditions in the laboratory were as described in Section 2.2. Each treatment consisted of 5 replicates. Plants were grown for 15 DAI then harvested to check for colonization on soybean hosts by fluorescence compound microscope (Olympus, SZX7).

### 2.7. Co-Inoculation Effect of S141 and Plant Growth Hormones on Soybean–Bradyrhizobium Symbiosis

Bacterial cell preparation was carried out as described in Section 2.1. Leonard’s jar experiments were performed as described in Section 2.2. The experiment was conducted to evaluate the co-inoculation effects of USDA110 with S141 or plant growth hormone on soybean. The seedlings were inoculated separately with 1 mL of 10^6^ CFU mL^−1^ of S141 or USDA110 for the single inoculation and mixed in a ratio of 1:1 for co-inoculation treatment. The cell suspension was replaced by sterilized distilled water in the control treatment. The plants were watered regularly with N-free nutrient solution during the experiment [30]. When necessary, the N-free nutrient solution was supplemented with appropriate plant growth hormones: 1 μg mL^−1^ cytokinin (6-Benzylaminopurine (Sigma-Aldrich, St. Louis, MO, USA) [32] and 10 μg mL^−1^ auxin (Indole-3-Acetic Acid (Sigma-Aldrich) [33,34]. Plant growth conditions in the laboratory were as described in Section 2.2.

### 2.8. Gene Disruption Related to Plant Growth Hormones Biosynthesis Pathway in S141 

#### 2.8.1. Construction of Gene Related IAA Biosynthesis Pathway

The putative genes related to the IAA biosynthesis pathway were disrupted, and the verifications of S141 mutant strains are shown in Figure S9. The putative genes related to the IAA biosynthesis pathway including *dhaS*, *yhcX*, and *IPyAD* were disrupted (Figure S10). Those determining antibiotics resistance, flanked by the respective gene sequences, were constructed for appropriate gene cassettes to replace the respective wild-type genes. The *dhaS*, *yhcX*, and *IPyAD* genes of S141 were inactivated by replacement with erythromycin, kanamycin, and spectinomycin resistance genes, respectively. The DNA fragments including upstream and downstream regions of *dhaS*, *yhcX*, and *IPyAD* were amplified by PCR using S141 chromosomal DNA as a template with primers dhaS_uF/dhaS_uR, yhcX_uF/yhcX_uR, and IPyAD_uF/IPyAD_uR, respectively, in normal condition for the upstream fragment and dhaS_dF/dhaS_dR, yhcX_dF/yhcX_dR, and IPyAD_dF/IPyAD_dR, respectively, for the downstream fragment (Table S2). Another DNA fragment containing the erythromycin resistance gene of *B. subtilis* strain YNB100 [26], kanamycin resistance gene of *B. subtilis* strain TMO311 [25], and spectinomycin resistance gene of *B. subtilis* strain TMO310 [25] (Table 1) were amplified using primers Erm_F/Erm_R, Kan_F/Kan_R, and Spm_F/Spm_R, respectively (Table S2). The three fragments (upstream, downstream, and antibiotic resistance gene) of each gene (*dhaS*, *yhcX*, and *IPyAD)* were ligated by recombinant PCR using primers dhaS_uF_nest/dhaS_dR_nest, yhcX_uF_nest/yhcX_dR_nest, and IPyAD_uF_nest/IPyAD_dR_nest, respectively, to sandwich each antibiotic resistance gene between the upstream and downstream regions of *dhaS*, *yhcX*, and *IPyAD*. The recombinant PCR fragment was transformed into S141 by natural competent method conferring erythromycin, kanamycin, and spectinomycin resistance and yielding the new strains S141Δ*dhaS* (*dhaS::erm^r^*), S141Δ*yhcX* (*yhcX::kan^r^*), and S141Δ*IPyAD* (*IPyAD::spm^r^*), which were used as the mutant strains for the IAA biosynthesis pathway in this study.

#### 2.8.2. Construction of Gene Related Cytokinin Biosynthesis Pathway

In S141, the gene related to cytokinin biosynthesis pathway including *IPT* (a gene coding for isopentenyl transferase) and *IPI* (a gene coding for isopentenyl isomerase) [35] were also disrupted (Figure S12). The genes determining antibiotics resistance, flanked by the respective gene sequences, were constructed for appropriate gene cassettes to replace the respective wild-type genes. The *IPT* and *IPI* genes of S141 were inactivated by replacement with phleomycin and kanamycin resistance genes, respectively. The DNA fragments corresponded to upstream and downstream regions of *IPT* and *IPI* were amplified by PCR using S141 chromosomal DNA as a template with primers IPT_uF/IPT_uR and IPI_uF/IPI_uR, respectively, for the upstream fragment and IPT_dF/IPT_dR and IPI_dF/IPI_dR, respectively, for the downstream fragment (Table S2). Another DNA fragment containing the phleomycin resistance gene of *B. subtilis* strain TSU007 and kanamycin resistance gene of *B. subtilis* strain TMO311 (Table 1) were amplified using primers Phl_F/Phl_R and Kan_F/Kan_R, respectively (Table S2). The recombinant PCR was applied to ligate three fragments (upstream, downstream, and antibiotic resistance gene) of each gene: *IPT* and *IPI* using primers IPT_uF_nest/IPT_dR_nest and IPI_uF_nest/IPI_dR_nest, respectively, to sandwich each antibiotic resistance gene between the upstream and downstream regions of *IPT* and *IPI*. The recombinant PCR fragment was transformed into S141 conferring phleomycin and kanamycin resistance and yielding the new strain, S141Δ*IPT* (*IPT::erm^r^*) and S141Δ*IPI* (*IPI::kan^r^*), which were used as the mutant strains for the cytokinin biosynthesis pathway.

Leonard’s jar experiments are described in Section 2.2. The experiments were conducted to evaluate the co-inoculation effect of S141, its mutant strains, and complementation by auxin (Indole-3-Acetic Acid (Sigma-Aldrich) on soybean–*Bradyrhizobium* symbiosis. S141 mutant strains were grown on LB medium broth at 30 °C. The medium was supplemented with appropriate antibiotics: 1 μg mL^−1^ erythromycin, 10 μg mL^−1^ kanamycin, 100 μg mL^−1^ spectinomycin, and 8 μg mL^−1^ phleomycin when necessary. The seedlings were inoculated separately with 1 mL of 10^6^ CFU mL^−1^ of each strain for the single inoculation and mixed in a ratio of 1:1 for co-inoculation treatment. The cell suspension was replaced by sterilized distilled water in the control treatment. Different amounts of auxin in the form of Indole-3-Acetic Acid were added into the S141 IAA-related mutant strains to complement the level of auxin produced by the S141 wild type. Colorimetric quantification of the IAA amounts present in culture filtrates of strains S141, S141Δ*dhaS*, S141Δ*yhcX*, and S141Δ*IPyAD* revealed IAA amounts of 5.13, 1.55, 1.48 and 0.20 µg mL^−1^, respectively. Therefore, IAA at concentrations of 3.58, 3.65, and 4.93 µg mL^−1^ was added into sterilized distilled water containing N-free nutrient solution of treatments inoculated with S141Δ*dhaS*, S141Δ*yhcX*, and S141Δ*IPyAD*, respectively. Each treatment consisted of five replicates. Plants were grown for 45 DAI, then harvested, and nodule number, nodule dry weight, shoot dry weight, root dry weight recorded, and nitrogenase activity measured using ARA [2]. The total plant dry weights were recorded, the tissues were placed in a hot air oven at 65 °C for 4 days before weighing (modified from [2]).

#### 2.8.3. IAA Production Assay

The IAA production of S141 and S141 IAA-related mutant strains were grown in LB medium broth supplement with 0.1% tryptophan for 48 h at 30 °C. After incubation, the IAA production was colorimetrically determined as described by Costacurta et al. [36]. The standard was prepared using pure indole-3-acetic acid (Sigma-Aldrich).

### 2.9. The Statistical Analysis

To test the data from each experiment, the normality and homogeneity of variances for each variable were first submitted and then analysis of variance (ANOVA). The post hoc test (Duncan’s multiple range test at *p ≤ 0.05* [37]) was used as a multiple comparison procedure as described by Duncan (1955) using SPSS^®^ software for WINDOWS^TM^, Version 14.0 (SPSS, Chicago, IL, USA) when confirming a statistically significant value in the F-test (*p ≤ 0.05*).

## 3. Results

### 3.1. Co-Inoculation of S141 Enhanced N_2_ Fixation Efficiency in Soybean–Bradyrhizobium Symbiosis

**Figure 1:**
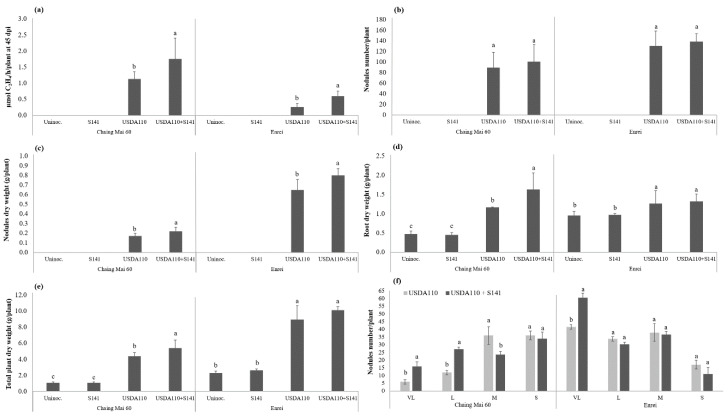
Plant growth and symbiotic parameters of soybean cultivar Chiang Mai 60 and Enrei by co-inoculation with S141 and USDA110 at 45 DAI: (**a**) nitrogenase activity was determined using the acetylene reduction assay; (**b**) nodule numbers per plant; (**c**) nodule dry weight; (**d**) root dry weight; (**e**) total plant dry weight; and (**f**) nodule size separation. Significance at *p* ≤ 0.05 is indicated by mean standard error bars (n = 8) of soybean cultivar Chiang Mai 60 and (n = 6) of soybean cultivar Enrei.

Differentiation in plant growth parameters of soybean cultivar Chiang Mai 60 (CM60) at 45 DAI following single inoculation and co-inoculation is presented in Figure 1. At 45 DAI, S141 showed significant increases in nitrogen fixation activity, nodule, root, and total plant dry weight when co-inoculated with strain USDA110 compared to the single inoculation of USDA110 (*p* < 0.05). The co-inoculation of S141 with USDA110 showed the highest nitrogen-fixing efficiency, nodule, root, and total plant dry weight, which are higher than single inoculation by 55.8%, 29.4%, 40.5%, and 22.9%, respectively. Nodule number per plant did not differ between the two treatments (Figure 1b), but nodule dry weight (Figure 1c) did show a significant difference which implies the sizes of the nodule were different. Nodules were classified into four groups by measuring the longest diameter (Table 2). The nodule size separation demonstrated a significant difference between single and co-inoculation (Figure 1f). The co-inoculation of S141 with USDA110 showed the highest number of very large (VL) size and large (L) size nodules when compared with single inoculation, whereas the single inoculation of USDA110 showed the highest number of small (S) size nodule when compared with co-inoculation treatment.

The variations in plant growth and symbiotic parameters of soybean cultivar Enrei at 45 DAI as a consequent of single inoculation and co-inoculation are also shown in Figure 1. The comparison between co-inoculation of S141 with USDA110 and single inoculation showed the highest nitrogen-fixing efficiency and nodule dry weight in co-inoculation treatment. In addition, co-inoculation of S141 with USDA110 also did not show a significant difference between single inoculation and co-inoculation of nodule number per plant (Figure 1b), but the nodule dry weight (Figure 1c) showed a significant difference indicating that sizes of the nodules are different, which is consistent with the result of CM60. The nodule size separation demonstrated a significant difference between single inoculation and co-inoculation (Figure 1f). The co-inoculation of S141 with USDA110 showed the highest number of VL size nodule, which increased up to 45.7% when compared with single inoculation. 

### 3.2. Influence of Biological Molecules Secreted from S141 on Soybean–Bradyrhizobium 

**Figure 2:**
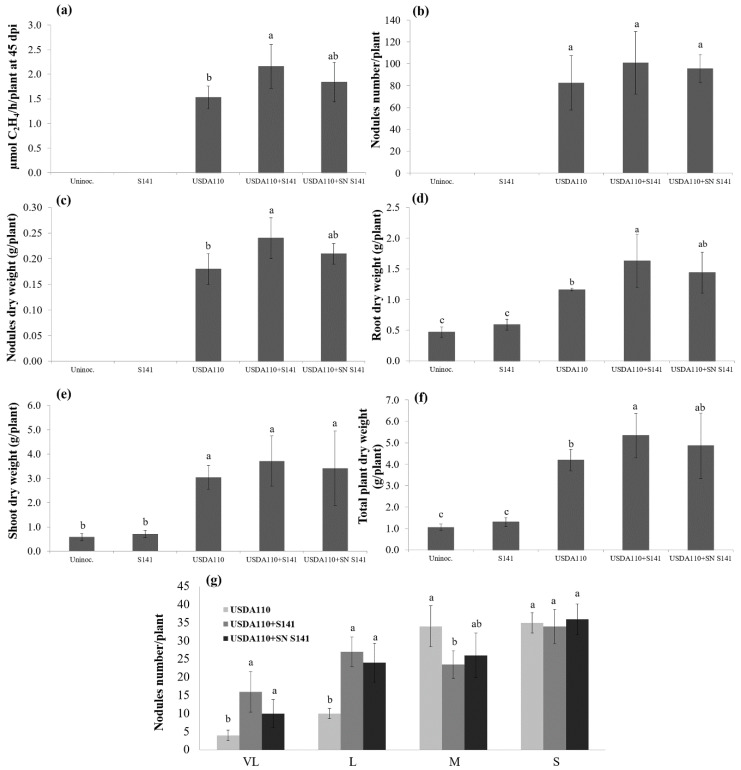
Plant growth and symbiotic parameters of soybean cultivar Chiang Mai 60 by co-inoculation with cells or supernatants of S141 and USDA110 at 45 DAI: (**a**) nitrogenase activity was determined by the acetylene reduction assay; (**b**) nodule numbers per plant; (**c**) nodule dry weight; (**d**) root dry weight; (**e**) shoot dry weight; (**f**) total plant dry weight; and (**g**) nodule size separation. Significance at *p ≤ 0.05* is indicated by mean standard error bars (n = 10).

S141 and its supernatant increased nitrogen fixation activity, nodule, root, shoot, and total plant dry weight when co-inoculated with USDA110 over the single inoculation of USDA110 on soybean cultivar CM60 at 45 DAI (*p* < 0.05). Variations in plant growth parameters of soybean at 45 DAI based on single inoculation and co-inoculation are illustrated in Figure 2. The co-inoculation of either cells or supernatant of S141 with USDA110 showed the highest nitrogen-fixing efficiency, nodule dry weight, root dry weight, and total plant dry weight when compared with single USDA110 inoculation and control, which are higher than single inoculation by 20.3–41.2%, 16.7–33.3%, 24.1–40.5%, and 15.7–27.1%, respectively. Meanwhile, co-inoculation of USDA110 with supernatant of S141 showed no significant difference with a single inoculation of USDA110 in terms of nitrogen-fixing efficiency, nodule dry weight, root dry weight, and total plant dry weight on soybean–Bradyrhizobium symbiosis. The results reveal that co-inoculation of USDA110 with supernatant of S141 tends to increase nitrogen-fixing efficiency, nodule dry weight, root dry weight, and total plant dry weight at 45 DAI. Moreover, co-inoculation of neither cells nor supernatant of S141 with USDA110 also showed a significant difference between single inoculation and co-inoculation of nodule number per plant (Figure 2b), but nodule dry weight (Figure 2c) demonstrated a significant difference which indicates the sizes of the nodule are different. This corresponds with the result of soybean cultivars CM60 and Enrei. The nodule size separation revealed a significant difference between single inoculation and co-inoculation in both cells and supernatant of S141 (Figure 2g and Figure S1b). The co-inoculation of either cells or supernatant of S141 with USDA110 showed the highest number of VL size and L size nodules, which increased 150–300% and 140–170%, respectively when compared with single inoculation. Besides, the single inoculation of USDA110 showed the highest number of S size nodule when compared with co-inoculation treatments because of not significantly different in nodule number per plant (Figure 2b).

Furthermore, S141 and its supernatant were evaluated for their plant growth promotion using soybean cultivars CM60 by co-inoculation with *B. diazoefficiens* CB1809. CB 1809 has been selected for higher efficiency of nitrogen fixation and higher adaptation to tropical soils. CB 1809 is the commercial strain that is broadly applied to soybean crops in the tropics, and it is known for its high efficiency in fixing nitrogen, but less competitiveness. The strain has been used in commercial inoculants in Brazil since 1992 [38]. S141 and its supernatant exhibited the ability to promote one or more plant parameters when co-inoculated with CB1809 over the single inoculation of CB1809 on soybean at 45 DAI (*p < 0.05*). The variations in plant growth parameters of soybean at 45 DAI based on single inoculation and co-inoculation are presented in Figure S2. The co-inoculation of either with cells or supernatant of S141 with CB1809 showed the highest nodule dry weight, root dry weight, shoot dry weight, and total plant dry weight when compared with single inoculation and control. On the other hand, the co-inoculation of CB1809 with supernatant of S141 also showed no significant difference when compared with single inoculation of CB1809 in terms of nodule dry weight, root dry weight, shoot dry weight, and total plant dry weight on soybean–*Bradyrhizobium* symbiosis. The results indicate that co-inoculation of CB1809 with the supernatant of S141 tends to increase nodule dry weight, root dry weight, and total plant dry weight on soybean–*Bradyrhizobium* symbiosis at 45 DAI, but the results show indistinctly nodule dry weight, root dry weight, and total plant dry weight when compared with co-inoculation of USDA110 with supernatant of S141. The co-inoculation of either cells or supernatant of S141 with CB1809 also did not show a significant difference between single inoculation and co-inoculation in terms of nodule number per plant (Figure S2a) which was consistent with co-inoculation of cells or supernatant of S141 with USAD110, but nodule dry weight showed a significant difference (Figure S2b), indicating that sizes of the nodule are different. The nodule size separation demonstrated a significant difference between single inoculation and co-inoculation of both cells and supernatant of S141 (Figure S3a,b). The co-inoculation of either cells or supernatant of S141 with CB1809 showed the highest number of VL size nodule, which increased 59.3–74.1% when compared with single inoculation, while L, M, and S size nodules of co-inoculation between supernatant of S141 with CB1809 did not show a significant difference when compared with single inoculation. 

### 3.3. Effect of Biological Molecules Secreted from S141 on Inoculation Dose of Bradyrhizobium 

There were differences among USDA110 doses for all soybean growth parameters including nodule numbers, nodule dry weight, root dry weight, shoot dry weight, and total plant dry weight (Figure S4). The results of the single inoculation of USDA110 were not different when compared with co-inoculation in all inoculant doses tested (10^3^–10^8^ CFU mL^−1^) on soybean nodule numbers, root dry weight, shoot dry weight, and total plant dry weight. All inoculant doses tested (10^3^–10^8^ CFU mL^−1^) of single inoculation of USDA110 or co-inoculation treatments produced higher root dry weight, shoot dry weight, and total plant dry weight than those obtained from uninoculated control treatments. In the case of nodule dry weight (Figure S4d), co-inoculation treatments of USDA110 at inoculant doses tested 10^5^–10^8^ CFU mL^−1^ produced higher nodule dry weight than 10^3^–10^4^ CFU mL^−1^, it was indicated that co-inoculation can help the soybean to produced L size nodules as the result of increasing nitrogen-fixing efficiency.

**Figure 3:**
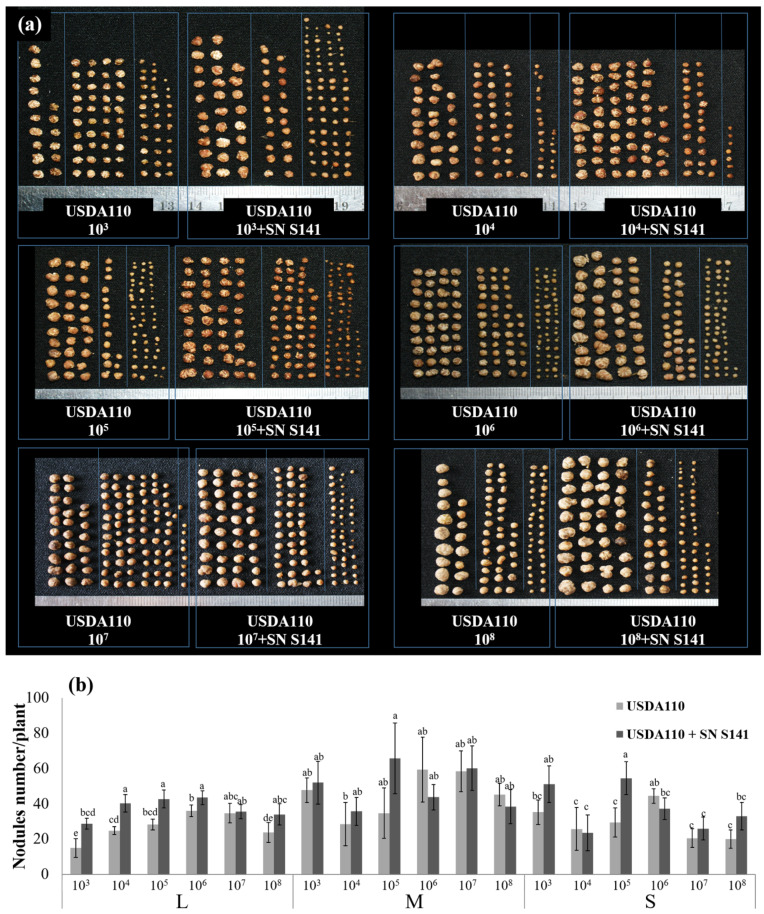
The nodule size separation of soybean cultivar Chiang Mai 60 by single inoculation and co-inoculation of supernatant of S141 with various doses of USDA110 at 45 DAI: (**a**) photograph of soybean nodule size separation of single inoculation and co-inoculation of supernatant of S141 with various doses of USDA110; and (**b**) soybean nodule size separation of single inoculation and co-inoculation with supernatant of S141 with various doses of USDA110. The number at the x-axis symbolized the varied inoculation doses of USDA110 are 10^3^, 10^4^, 10^5^, 10^6^, 10^7^, and 10^8^ CFU mL^−1^. Significance at *p ≤ 0.05* is indicated by mean standard error bars (n = 5).

Remarkably, the nodule size separation (Figure 3a,b) of co-inoculation treatments with supernatant of S141 and USDA110 at inoculant doses tested 10^3^–10^6^ CFU mL^−1^ produced the highest number of L size nodules when compared with single inoculation in each inoculant doses tested while increasing inoculant doses tested 10^7^–10^8^ CFU mL^−1^, the number of L size nodules did not show a significant difference when compared with the numbers of nodule derived from single inoculation and co-inoculation treatments. For the numbers of M and S size nodules, it also showed no difference when compared with the numbers of nodule derived from single inoculation and co-inoculation treatments in each inoculant doses tested. 

### 3.4. Co-Inoculation Effect of S141 on Bradyrhizobium Competition

**Figure 4:**
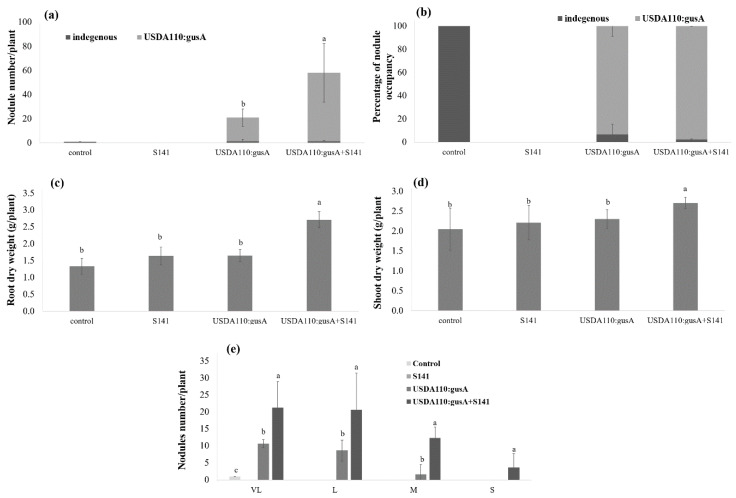
Plant growth parameters of soybean cultivar Chiang Mai 60 by co-inoculation with S141 and GUS-tagged USDA110 at 45 DAI: (**a**) nodule numbers per plant; (**b**) percentage of nodule occupancy; (**c**) root dry weight; (**d**) shoot dry weight; and (**e**) nodule size separation. Significance at *p ≤ 0.05* is indicated by mean standard error bars (n = 5).

To demonstrate soybean–*Bradyrhizobium* symbiosis competition of co-inoculation by bradyrhizobial strain GUS-tagged USDA110 and S141 with indigenous *Bradyrhizobium*, the soybean–*Bradyrhizobium* symbiosis competitions were conducted in the pot. Tap-root nodules were harvested 45 days after planting, and nodule occupancies were determined for the bradyrhizobial strain originally applied. In soybean, there were differences among single inoculation and co-inoculation for all soybean growth parameters including nodule numbers, root dry weight and shoot dry weight (Figure 4). The results of co-inoculation of GUS-tagged USDA110 and S141 showed a significant difference in nodule numbers, root dry weight, and shoot dry weight compared with single inoculation treatments and control. Interestingly, co-inoculation of GUS-tagged USDA110 and S141 demonstrated the highest nodule numbers (Figure 4a); most of the nodules were formed by GUS-tagged USDA110 when compared with single inoculation, but the percentage of nodule occupancy did not show a significant difference (Figure 4b) when compared with single inoculation. These results suggest the co-inoculation of GUS-tagged USDA110 and S141 consistently altered nodulation among certain combinations of bradyrhizobial strains. This alteration typically reflected the enhancement of nodulation by the co-inoculation of S141 with GUS-tagged USDA110. The nodule size separation indicated a significant difference between single inoculation and co-inoculation (Figure 4e). The co-inoculation of S141 with GUS-tagged USDA110 exhibited the highest nodule number in all sizes of nodules when compared with single inoculation because co-inoculation of S141 with GUS-tagged USDA110 showed a significant difference of nodule number when compared with single inoculation.

### 3.5. Colonization of S141 on Soybean–Bradyrhizobium Symbiosis

The green fluorescent protein (GFP) was constructed into S141, and GFP gene was activated in S141 by insertion to *tuf* promoter gene (Figure S5), which was used as the GFP-tagged strain for the colonization on soybean–*Bradyrhizobium* symbiosis (Figure S6). The colonization of S141 on soybean–*Bradyrhizobium* symbiosis is demonstrated in Figure S7. The results show S141 colonizes the nodule and root surface of soybean. These results confirm that S141 associatively localized at the nodule and root surface.

### 3.6. Effect of S141 and Plant Growth Hormone on Soybean–Bradyrhizobium Symbiosis 

**Figure 5:**
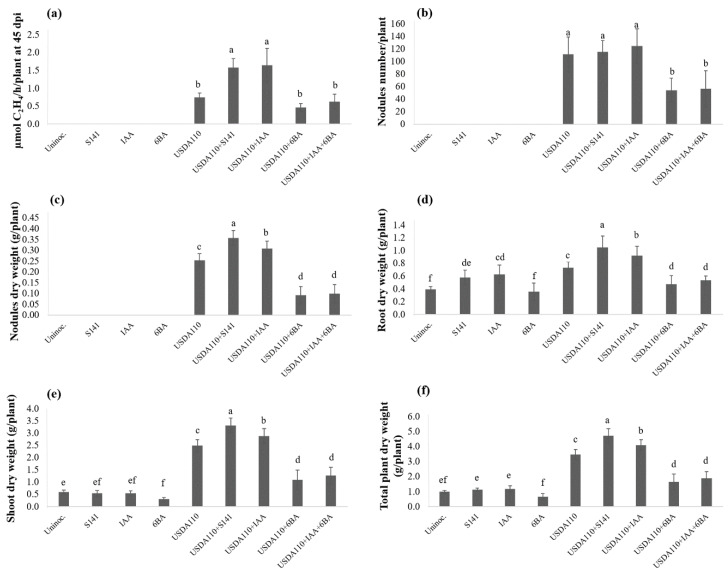
Plant growth parameters of soybean cultivar Chiang Mai 60 by co-inoculation with S141 and USDA110 compared with single inoculation supplemented with Indole-3-Acetic Acid and/or 6-Benzylaminopurine at 45 DAI: (**a**) nitrogenase activity was determined using the acetylene reduction assay; (**b**) nodule numbers per plant; (**c**) nodule dry weight; (**d**) root dry weight; (**e**) shoot dry weight; and (**f**) total plant dry weight. Significance at *p ≤ 0.05* is indicated by mean standard error bars (n = 8).

**Figure 6:**
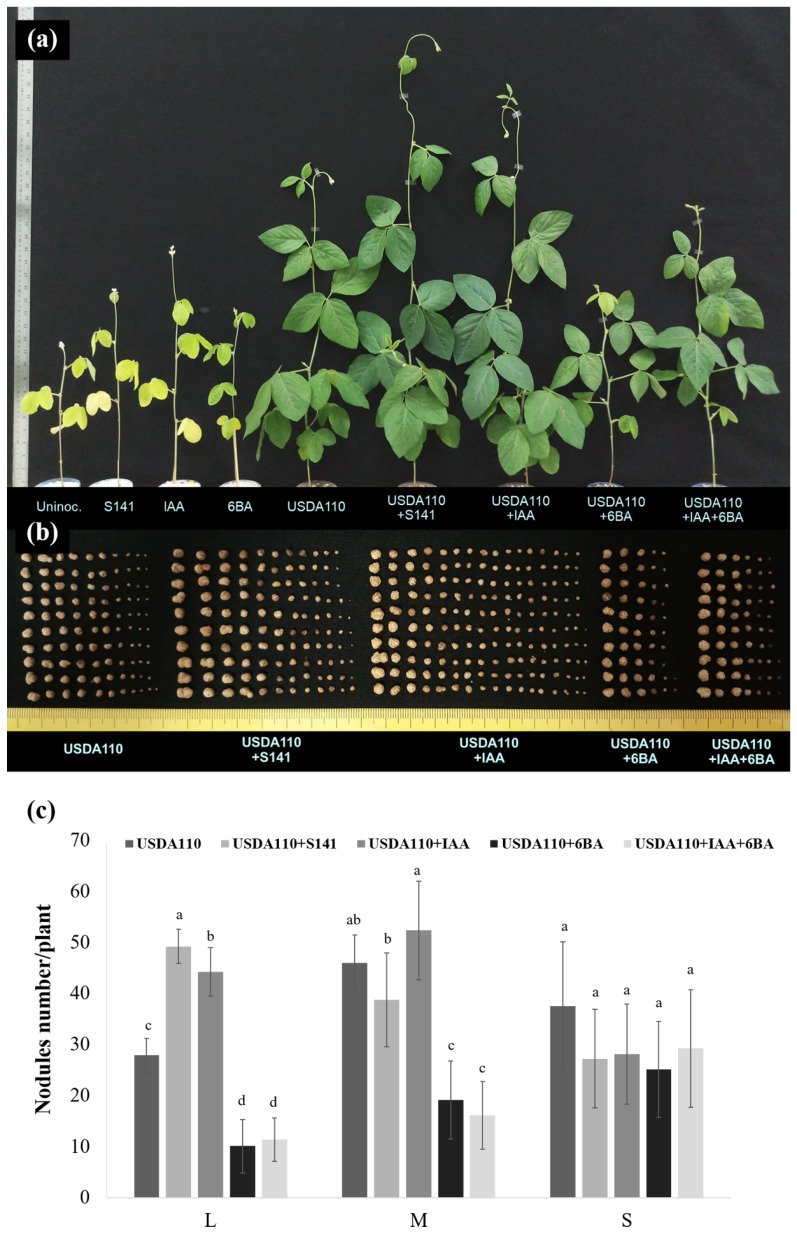
Soybean plant growth and the nodule size separation of soybean cultivar Chiang Mai 60 by co-inoculation with S141 and USDA110 and/or supplemented with IAA and/or 6BA at 45 DAI: (**a**) soybean growth under Leonard’s jar experiments; (**b**) photograph of soybean nodule; and (**c**) nodule size separation. Significance at *p ≤* 0.05 is indicated by mean standard error bars (n = 8).

**Figure 7:**
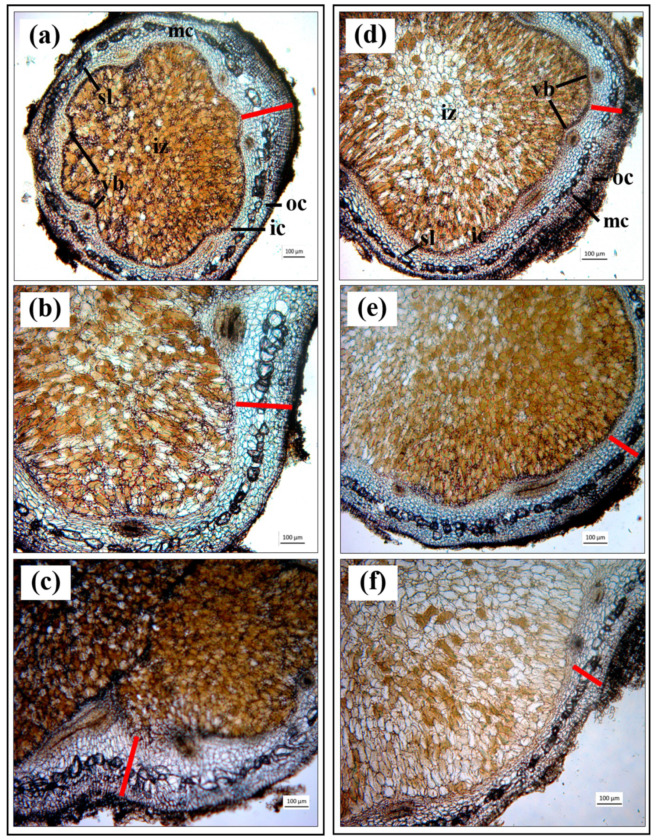
Comparison of soybean nodule cross section under light compound microscope between single inoculation of USDA110 and co-inoculation of USDA110 with S141 at 45 DAI: (**a**) small nodule of single inoculation; (**b**) medium nodule of single inoculation; (**c**) large nodule of single inoculation; (**d**) small nodule of co-inoculation; (**e**) medium nodule of co-inoculation; and (**f**) large nodule of co-inoculation. iz, infected zone; ic, inner cortex; mc, middle cortex; oc, outer cortex; vb, vascular bundles; sl, sclerid layer. Red bar indicates length of cortex zone.

Variations in plant growth parameters of soybean at 45 DAI as a consequent of single inoculation, single inoculation supplemented with IAA and/or 6-BA and co-inoculation are presented in Figure 5. The co-inoculation of S141 with USDA110 showed the highest nitrogen-fixing efficiency, nodule dry weight, root dry weight, shoot dry weight, and total plant dry weight when compared with all inoculation treatments and control. At the same time, nitrogen-fixing efficiency (Figure 5a) was not different between co-inoculation of USDA110 with S141 and single inoculation of USDA110 supplemented with IAA, but it showed a significant difference with a single inoculation of USDA110 supplemented with 6-BA and 6-BA plus IAA treatments. In the case of nodule dry weight (Figure 5c), root dry weight (Figure 5d), shoot dry weight (Figure 5e), and total plant dry weight (Figure 5f), the results display the same pattern, which is the highest in co-inoculation of S141 with USDA110 followed by a single inoculation of USDA110 supplemented with IAA, single inoculation of USDA110, single inoculation of USDA110 supplemented with IAA plus 6-BA and single inoculation of USDA110 supplemented with 6-BA, respectively. The co-inoculation of S141 with USDA110 also did not show a significant difference in nodule dry weight with single inoculation of USDA110 and single inoculation of USDA110 supplemented with IAA, but it showed a significant difference with single inoculation of USDA110 supplemented with 6-BA and 6-BA plus IAA treatments. Meanwhile, nodule dry weight demonstrated a significant difference (Figure 5c), indicating that the sizes of the nodule are different. The nodule size separation showed a significant difference between single inoculation and co-inoculation, as presented in Figure 6. The co-inoculation of S141 with USDA110 showed the highest nodule number of L size nodules when compared with single inoculation followed by a single inoculation of USDA110 supplemented with IAA, single inoculation of USDA110, single inoculation of USDA110 supplemented with IAA plus 6-BA, and single inoculation of USDA110 supplemented with 6-BA, respectively. In addition, the single inoculation of USDA110 supplemented with IAA displayed the highest nodule number of medium (M) size nodules when compared with all inoculation treatments. The nodule size separation was changed to the percentage of each nodule size. Consideration the percentage of each nodule size regardless of nodule number demonstrated the significantly different between single inoculation and co-inoculation, as presented in Figure S8. The co-inoculation of S141 with USDA110 and single inoculation of USDA110 supplemented with IAA performed the highest percentage of nodule number of L size nodule and showed a significant difference with single inoculation of USDA110, single inoculation of USDA110 supplemented with IAA plus 6-BA, and single inoculation of USDA110 supplemented with 6-BA, but the nodule number in M size nodule was not different in all treatments. In contrast, the nodule number of S size nodule showed the highest in the single inoculation of USDA110 supplemented with IAA plus 6-BA and single inoculation of USDA110 supplemented with 6-BA followed by a single inoculation of USDA110, co-inoculation of S141 with USDA110, and single inoculation of USDA110 supplemented with IAA, respectively. The cross section of nodules from co-inoculation of USDA110 with S141 and single inoculation of USDA110 in each size is depicted in Figure 7. Co-inoculation of USDA110 with S141 nodules cross-section showed a thinner layer of the cortex and larger area of the infection zone than the nodules of single inoculation. It is obvious that IAA might play an important role in cortex cell elongation and proliferation. 

### 3.7. Effect of Plant Growth Hormone Biosynthesis in S141 on Soybean–Bradyrhizobium Symbiosis 

The result of IAA production in S141 and S141 IAA-related mutant strains are summarized in Figure S11. The IAA amounts presented in culture filtrates were quantified including strains S141Δ*dhaS*, S141Δ*yhcX*, and S141Δ*IPyAD* by the colorimetrically methodology revealed reduced amounts of IAA. The S141Δ*IPyAD* mutant strain formed only 3.83%, while the strain bearing the mutation S141Δ*yhcX* produced 28.94% and S141Δ*dhaS* produced 30.22% of the IAA amount produced in wild type.

**Figure 8:**
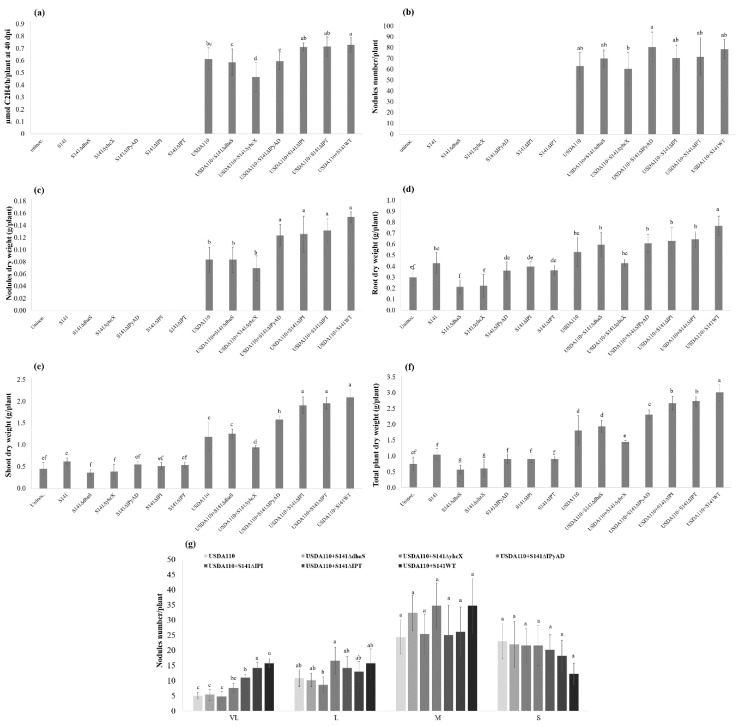
Plant growth parameters of soybean cultivar Chiang Mai 60 by co-inoculation with USDA110 and S141 or S141 mutant strains compared with single inoculation at 45 DAI: (**a**) nitrogenase activity was determined using the acetylene reduction assay; (**b**) nodule numbers per plant; (**c**) nodule dry weight; (**d**) root dry weight; (**e**) shoot dry weight; (**f**) total plant dry weight; and (**g**) nodule size separation. Significance at *p ≤* 0.05 is indicated by mean standard error bars (n = 8).

The results show that S141 could significantly promote one or more plant parameters when co-inoculated USDA110 over the single inoculation and co-inoculation with S141 mutant strains on soybean at 45 DAI (*p < 0.05*). Variations in plant growth parameters of soybean at 45 DAI as a consequence of single inoculation or co-inoculation with S141 mutant strains are presented in Figure 8. The co-inoculation of S141 with USDA110 showed the highest nitrogen-fixing efficiency, nodule dry weight, root dry weight, shoot dry weight, and total plant dry weight compared with all inoculation treatments and control. The nitrogen-fixing efficiency (Figure 8a) was not different in co-inoculation with USDA110 and S141, co-inoculation of USDA110 and S141Δ*IPT*, and co-inoculation of USDA110 and S141Δ*IPI* but showed a significant difference with single inoculation of USDA110, and co-inoculation of USDA110 with mutant strains S141Δ*dhaS* and S141Δ*IPyAD*, and showed the lowest nitrogen-fixing efficiency on co-inoculation of USDA110 with S141Δ*yhcX*. In the case of nodule dry weight (Figure 8c), it demonstrated the highest value when co-inoculated USDA110 with S141. Co-inoculation of USDA110 with S141Δ*IPT*, co-inoculation of USDA110 with S141Δ*IPI*, and co-inoculation of USDA110 with S141Δ*IPyAD* showed significant difference when compared with co-inoculation of USDA110 with S141Δ*yhcX*, co-inoculation of USDA110 with S141Δ*dhaS*, and single inoculation of USDA110. For root dry weight (Figure 8d), it showed the highest value when co-inoculated USDA110 with S141 followed by co-inoculation of USDA110 with all of mutant strains and single inoculation of USDA110, and the lowest numbers were found in S141Δ*dhaS*, S141Δ*yhcX*, and control. The shoot dry weight (Figure 8e) and total plant dry weigh (Figure 8f) showed the same pattern: the highest values were found when co-inoculated S141 with USDA110 followed by co-inoculation of USDA110 with both cytokinin related mutant strains (S141Δ*IPT* and S141Δ*IPI*), co-inoculation of USDA110 with S141Δ*IPyAD*, co-inoculation of USDA110 with S141Δ*dhaS*, single inoculation of USDA110, and co-inoculation of USDA110 with S141Δ*yhcX*, and the lowest in S141Δ*dhaS* and S141Δ*yhcX*, respectively. The co-inoculation of S141 with USDA110 also did not show a significant difference in nodule dry weight with single inoculation of USDA110 and co-inoculation of USDA110 with S141Δ*IPT*, S141Δ*IPI*, S141Δ*IPyAD*, and S141Δ*dhaS*, but it displayed a significant difference with co-inoculation of USDA110 with S141Δ*yhcX*. Meanwhile, nodule dry weight showed a significant difference (Figure 8c), which indicated that sizes of the nodule are different. The nodule size separation showed a significant difference between single inoculation and co-inoculation, as illustrated in Figure 8g and Figure S13. The co-inoculation of S141 with USDA110 showed the highest nodule number of VL size nodules similarly with co-inoculation of USDA110 with S141Δ*IPT*, while co-inoculations of USDA110 with S141Δ*IPI*, S141Δ*IPyAD*, S141Δ*yhcX*, and S141Δ*dhaS* reduced nodule number of VL size nodules. Moreover, co-inoculation of USDA110 with S141Δ*yhcX* also reduced nodule number of L size nodules. 

**Figure 9:**
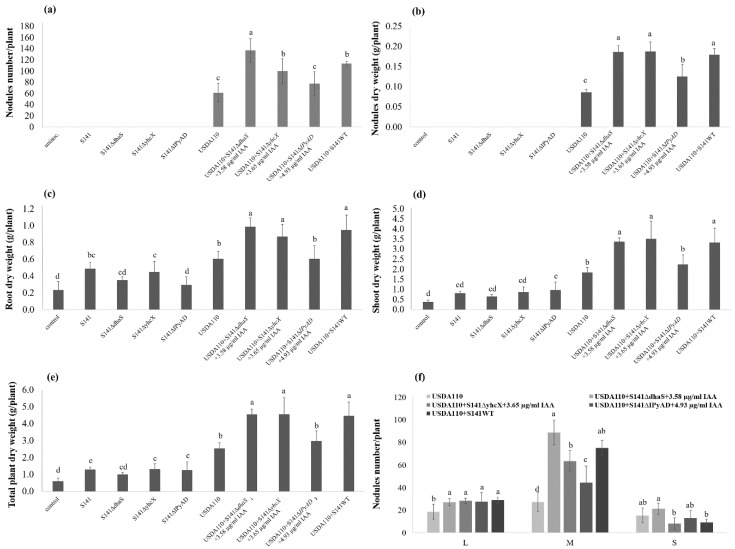
Plant growth parameters of soybean cultivar Chiang Mai 60 by co-inoculation with USDA110 and S141 or S141 mutant strains complemented with Indole-3-Acetic Acid compared with single inoculation at 45 DAI: (**a**) nodule numbers per plant; (**b**) nodule dry weight; (**c**) root dry weight; (**d**) shoot dry weight; (**e**) total plant dry weight; and (**f**) nodule size separation. Significance at *p ≤* 0.05 is indicated by mean standard error bars (n = 6).

The effect of co-inoculation between USDA110 and S141 or S141 IAA-related mutant strains including S141Δ*dhaS*, S141Δ*yhcX*, and S141Δ*IPyAD* complemented by the addition of 3.58, 3.65, and 4.93 µg mL^−1^ IAA, respectively, on soybean–*Bradyrhizobium* symbiosis in comparison with single inoculation was demonstrated. The results show that S141 could significantly promote one or more plant parameters when co-inoculated with USDA110 over the single inoculation on soybean at 45 DAI (*p <* 0.05). Variations in plant growth parameters of soybean at 45 DAI as a consequence of single inoculation and co-inoculation are presented in Figure 9. The co-inoculation of USDA110 and S141Δ*dhaS* complemented with 3.58 µg mL^−1^ IAA showed the highest nodule number followed by co-inoculation of USDA110 and S141 wild type, co-inoculation of USDA110 and S141Δ*yhcX* complemented with 3.65 µg mL^−1^ IAA, co-inoculation of USDA110 and S141Δ*IPyAD* complemented with 4.93 µg mL^−1^ IAA, and single inoculation of USDA110, respectively (Figure 9a). In the case of nodule dry weight (Figure 9b), root dry weight (Figure 9c), shoot dry weight (Figure 9d), and total plant dry weight (Figure 9e), the results display the same pattern: the highest in co-inoculation of USDA110 and S141 wild type, co-inoculation of USDA110 and S141Δ*dhaS* complemented with 3.58 µg mL^−1^ IAA, and co-inoculation of USDA110 and S141Δ*yhcX* complemented with 3.65 µg mL^−1^ IAA, while co-inoculation of USDA110 and S141Δ*IPyAD* complemented with 4.93 µg mL^−1^ IAA showing higher nodule dry weight than single inoculation of USDA110 when compared with all inoculation treatments and control. The nodule dry weights were significantly different (Figure 9b), indicating that sizes of the nodule are different. The nodule size separation showed a significant difference between single inoculation and co-inoculation (Figure 9f and Figure S14). The co-inoculation of USDA110 with S141 or S141 IAA-related mutant strains (S141Δ*dhaS*, S141Δ*yhcX*, and S141Δ*IPyAD*) complemented with IAA showed the highest nodule number of L size nodules when compared with single inoculation. Moreover, the co-inoculation USDA110 with S141 or S141 IAA-related mutant strains (S141Δ*dhaS*, S141Δ*yhcX*, and S141Δ*IPyAD*) complemented with IAA also displayed the highest nodule number of M size nodules when compared with single inoculation.

## 4. Discussion

In this study, S141 demonstrated significant capability in promoting nitrogen-fixing efficiency, nodule number, nodule dry weight, size of nodules and total plant dry weight with soybean–*Bradyrhizobium* symbiosis by co-inoculation with *B. diazoefficiens* USDA110. Furthermore, co-inoculation with the supernatant of S141 with USDA110 produced similar results to those with the cells of S141. The co-inoculation of either cells or supernatant of S141 with USDA110 enhanced the number of VL size nodules and L size nodules when compared with single inoculation (Figure 2g). Nevertheless, co-inoculation of CB1809 with supernatant of S141 (Figure S2) tends to increase nodule dry weight, root dry weight, and total plant dry weight on soybean–*Bradyrhizobium* symbiosis, but these results were not clear when compared with the co-inoculation of USDA110 with supernatant of S141. It is possible that supernatant of S141 may be more compatible with USDA110 than CB1809, and therefore they are able to enhance soybean growth better than CB1809. The multiple strains are synergistic for plants [39], but, in some cases, mixed inoculations in plants performed worse than expected [40]. 

The effective inoculation doses of *B. diazoefficiens* USDA110 on soybean were 10^6^–10^8^ cells seed^−1^. However, the concentration of soybean inoculant USDA110 can be reduced when co-inoculation with cells or supernatant of S141 (Figure S4). These results suggest that co-inoculation of supernatant with S141 and USDA110 can promote soybean growth by enhancing the production of many L size nodules (Figure 3), resulting in increased nitrogen-fixation efficiency. Moreover, it implies that the concentration of cells of USDA110 can be reduced when co-inoculated with supernatant of S141. This finding greatly affects the seed inoculation process since the low number of rhizobia inoculation could be sufficient for nodulation when co-inoculated with PGPR. Based on this experiment, the inoculant can be improved using normal peat carriers with a mixture living cells of *B. diazoefficiens* and S141. In the case of S141 supernatant, the stability in peat carrier is still not observed. Co-inoculation of S141 cannot enhance the competitiveness of *Bradyrhizobium* on soybean nodulation, while co-inoculation of bradyrhizobial strain and S141 was significantly capable of promoting soybean growth by enhancing soybean nodulation in terms of increasing nodule number (Figure 4a), and producing the L size of nodules (Figure 4e) rendering increase of nitrogen-fixing efficiency and promoting soybean growth. Colonization of S141 around nodule and root surface of soybeans confirmed that S141 acts closely with rhizosphere as plant growth-promoting rhizobacteria (Figure S7). 

According to Prakamhang et al. [41], S141 produced amounts of IAA into 19.33 µg mL^−1^. The comparison with another PGPR *Bacillus* strain, *B. velezensis* FZB42 (*B. amyloliquefaciens* subsp. *plantarum* FZB42), found that S141 consisted of putative genes involved in indole-3-acetic acid (IAA) biosynthesis. They are summarized in Table S3. Interestingly, these three proteins including tryptophan transporter, Indole-3-glycerol phosphate, and tryptophan-rich sensory protein were found only in S141 genome, but they did not a presence in *B. velezensis* FZB42 genome. Furthermore, not only the IAA biosynthesis pathway was found in S141 but also the cytokinin biosynthesis pathway. The tRNA delta (2)-isopentenyl pyrophosphate (IPP) transferase domain was foretold by the genome database of S141 (protein_id BBA76310.1) that translated from *IPT* gene products, which is significant for the enzyme activity in cytokinin biosynthesis. Isopentenyl transferases (IPTs) implement the rate-limiting and first step in cytokinin biosynthesis, where an isopentenyl group is transferred to ATP, ADP, or AMP [42]. Besides, isopentenyl pyrophosphate isomerase (IPI) (protein_id BBA76708.1) was also found in S141 and is an isomerase that activates the conversion of the relatively inactive isopentenyl pyrophosphate (IPP) to the more active electrophile dimethylallyl pyrophosphate (DMAPP). This isomerization is an important step through the mevalonate pathway in the biosynthesis of isoprenoids and the MEP pathway [43]. Thus, these results suggest that S141 have multiple genes that are functionally related to cytokinin and auxin biosyntheses and play an important role in its capability to promote plant growth.

Interestingly, co-inoculation of USDA110 with S141 and single inoculation of USDA110 supplemented with IAA enhanced soybean–*Bradyrhizobium* symbiosis to produce L size of nodules (Figure 6b,c) as the result of increasing nitrogen-fixing efficiency. It seems that IAA, as well as IAA produced from S141, affected cell elongation of nodules. These results are consistent with those of Liu et al. [44], which indicate that both infection thread formation and nodule development are involved by the auxin signaling pathway. The auxin signaling initiates the infection thread, while cytokinin signaling is required for infection thread elongation. This leads to cell division and initiates nodule organogenesis induced by this symbiotic signal. Moreover, Zhang et al. [45] indicated that auxin is directly involved in the nodulation of peanut. Auxin and cytokinin are significant regulators for cortical cell differentiation and proliferation and are crucial for more nodule development. 

However, the commercial cytokinin 6-Benzylaminopurine (6-BA) does not promote soybean growth and nodulation in these experiments because it seems to have a high concentration of cytokinin and retards the growth of soybean–*Bradyrhizobium* symbiosis. Phytohormones such as auxins and cytokinins, which are produced by PGPR, can impact cell proliferation in the root structure by more production of root hairs and lateral roots with a subsequent enhancement of water and nutrient uptake [46,47]. Investigation of cytokinin by cytokinin overproduction and cytokinin deficient mutants confirmed that cytokinin is involved in the regulation of cell division activity of young leaves and shoot meristem [48]. Moreover, cytokinin can be produced by rhizobia, and a significant role in root nodule signaling symbiosis and nodule formation has been demonstrated [49]. Nodule development requires an appropriate level of cytokinin [44]. Besides, cytokinins in high concentrations obstruct cell proliferation and stimulate programmed cell death (PCD) in both *Arabidopsis thaliana* (L.) Heynh and carrot (*Daucus carota* L.) [50]. In addition, the exogenous application of cytokinins in soybean nodulation including trans-zeatin, 6-benzyl amino purine, and N6-(12-isopentenyl)-adenine via either petiole feeding or root drenching techniques showed that cytokinins are a significant regulator of cell differentiation and cell proliferation in plant development by increasing nodule numbers at low concentrations but reducing nodule numbers at higher concentrations [42]. Cytokinins can act as signal molecules that dominate plant growth and development, stimulate developmental processes, and affect cell division and cell cycle in plants [51]. The cross-section of nodules from the co-inoculation of USDA110 with S141 showed a thinner layer of the cortex and larger area of the infection zone than the nodules of single inoculation at all nodule sizes (Figure 7). Other experiments have indicated that hypernodulation mutant lines have thick cortical regions when compared with wild-type soybean nodules [52]. This may be caused by the signal from autoregulation of nodulation inhibiting cell division in further nodule development of wild-type soybean nodules, but not inhibiting it in hypernodulation mutant lines. IAA is also a positive regulator role in nodule development [53]. It is obvious that IAA might play an important role in cortex cell elongation and proliferation. This result is consistent with those of Liu et al. [44] who showed that auxin and cytokinin are significant regulators of cortical cell differentiation and proliferation and are important for further nodule development [50].The IAA amounts present in the culture filtrates of strains S141Δ*dhaS*, S141Δ*yhcX,* and S141Δ*IPyAD* revealed that all of S141 IAA-related mutant strains produced less than half the amount of IAA produced by the wild type (Figure S10 and Figure S11). The results suggest that all S141 IAA-related mutant strains are related to IAA biosynthesis pathway. However, Idris et al. [54] reported that IAA biosynthesis in *B. velezensis* strain E104 (Δ*dhaS*) was not affected implying no cooperation of the *dhaS* gene product in IAA biosynthesis. Although none of the IAA-related mutant strains completely abolished IAA production, co-inoculation of USDA110 with all of IAA-related mutant strains reduced nodule number of VL size nodules. Moreover, co-inoculation of USDA110 with S141Δ*yhcX* also reduced nodule number of L size nodules. It is most likely that *yhcX* may play an important role in IAA biosynthesis in soybean growth promotion (Figure 8g and Figure S13). The translated *yhcX* gene product is similar to nitrilase 2 of *Arabidopsis thaliana*, which activates the direct conversion of indole 3-acetonitrile to IAA [55]. The result of complementation by various amounts of auxin supplementation in co-inoculation between USDA110 and S141 or S141 IAA-related mutant strains indicated that the co-inoculation of USDA110 and S141 or S141 IAA-related mutant strains complemented with IAA enhanced to recover form of L size nodules when compared with single inoculation (Figure 9f and Figure S14). Moreover, the co-inoculation between USDA110 and S141 or S141 IAA-related mutant strains supplemented with IAA also displayed the highest number of M size nodules when compared with single inoculation treatment. Besides, the action inhibition and complementation experiments show that the signaling of auxin is involved in *Phomopsis liquidambari* mediated development of nodule, enhanced N_2_-fixation, and activation of the symbiotic gene. Moreover, the nodule physiology and morphology changes were stimulated by inhibition of auxin action compromised the *P. liquidambari*, including symbiosome and bacteroid density, development of the vascular bundle and malate concentrations, while the accumulation of starch granules induction in *P. Liquidambari* inoculated nodules [45]. However, the putative IAA acetyltransferase (*ysnE*) gene is localized within the cluster of the tryptophan biosynthesis gene of *A. brasilense*. The translated *ysnE* gene products stimulated the transfer of an acetyl group to a nitrogen atom on the acceptor molecule. IAA acetyltransferase is included in a widely distributed family of acetyltransferases and has been proposed to involve in tryptophan dependent IAA biosynthesis [54,56]. The results from this study strongly indicate the important pathway for IAA biosynthesis in S141 by the existence of a tryptophan-dependent pathway. However, IAA production may exist in S141 via insignificant tryptophan-independent pathways, because IAA production was not eradicated in the mutant strains. The mutation in both gene-related in tryptophan-independent and tryptophan dependent pathways might help to explain the presence of IAA production.

Considering cytokinin biosynthesis, co-inoculation of USDA110 with S141 enhanced soybean–*Bradyrhizobium* symbiosis to produce high nodule dry weight similar to co-inoculation of USDA110 with S141Δ*IPT* or S141Δ*IPI* as the result of increasing nitrogen-fixing efficiency, but co-inoculation of USDA110 with S141Δ*IPI* showed a reduction in the number of VL nodules. It seems likely isopentenyl pyrophosphate isomerase (IPI) may play an important role in controlling nodule size of soybean–*Bradyrhizobium* symbiosis.

## 5. Conclusions

*Bacillus velezensis* strain S141 could significantly promoted nitrogen-fixing efficiency, nodule number, nodule dry weight, size of nodules, and total plant dry weight with soybean–*Bradyrhizobium* symbiosis by co-inoculation with *Bradyrhizobium diazoefficiens* USDA110. The concentration of soybean inoculant USDA110 can be reduced when co-inoculated with cells or supernatant of S141. Besides, co-inoculation of bradyrhizobial strain and S141 was capable of promoting soybean growth by enhancing soybean nodulation in terms of increasing nodule number and increasing the size of nodules. S141 possesses multiple genes that are functionally related to auxin and cytokinin biosynthesis, which play key roles in its ability to promote plant growth. Exogenous IAA and IAA produced from S141 affected cell elongation and cell proliferation of nodules when co-inoculated with USDA110. This symbiotic signal induces cortex cell division and initiates nodule organogenesis. The disruption of putative genes related to IAA biosynthesis pathway suggested that all S141 IAA-related mutant strains affected IAA biosynthesis pathway. The co-inoculation of USDA110 with S141 enhanced soybean–*Bradyrhizobium* symbiosis to produce VL size of nodules as the result of increasing nitrogen-fixing efficiency. The IAA produced from S141 had the effect on cell elongation and cortical cell proliferation of nodules. Moreover, co-inoculation of USDA110 with S141Δ*yhcX* also reduced the number of L size nodules. This is the first report that *yhcX* may play a major role in IAA biosynthesis in *B. velezensis* as well as providing major impact on soybean growth promotion. In the case of cytokinin biosynthesis, the disruptions of genes related to cytokinin biosynthesis pathway including *IPT* and *IPI* genes were generated and applied in this study. However, co-inoculation of USDA110 with S141Δ*IPI* reduced nodule number of VL size nodules, indicating that isopentenyl pyrophosphate isomerase (IPI) may play an important role in controlling the nodule size of soybean–*Bradyrhizobium* symbiosis. Co-inoculation of USDA110 with S141 enhanced soybean–*Bradyrhizobium* symbiosis to produced high nodule dry weight, promoting soybean growth via greater nitrogen fixation. This is the first demonstration of the mechanism of *B. velezensis* S141 on soybean–*Bradyrhizobium* symbioses. Therefore, the strategy of enhancing soybean N_2_-fixation by S141 with *Bradyrhizobium* co-inoculation could be further developed for superior *Bradyrhizobium*–soybean inoculants.

## Figures and Tables

**Table 1:** Bacterial strains used in this study.

Bacterial Strains	Relevant Genotype or Description	Source or References
*Bradyrhizobium diazoefficiens* USDA110	Wild type	[8]
*B. diazoefficiens* CB1809	Wild type	DAR
*B. diazoefficiens* GUS-tagged USDA110	marked with mTn*5SSgusA20* (pCAM120); Sm^r^ Sp^r^	[8]
*Bacillus subtilis* 168:ytsJ:gfp	*ytsJ* *::gfp* *::erm^r^*	Kobe University
*B. subtilis* TSU077	*trpC2, epr* *::PrpsO* *-dam* *(Phle* *)*	Kobe University
*B. subtilis* TMO310	*trpC2 aprE::(spc lacI* P*spac-mazF)*	[25]
*B. subtilis* TMO311	*trpC2 aprE::(kan lacI* P*spac-mazF)*	[25]
*B. subtilis* YNB100	*trpc2 aprE::kan yhcT::(oriTLS20-F erm)* pLS20catΔ*oriT*	[26]
*Bacillus velezensis* S141	Wild type	[24]
*B. velezensis* S141:GFP	*tuf* *::gfp* *::phle^r^*	This study
*B. velezensis* S141Δ*dhaS*	*dhaS* deletion, Δ*dhaS::erm^r^*	This study
*B. velezensis* S141Δ*yhcX*	*yhcX* deletion, Δ*yhcX::kan^r^*	This study
*B. velezensis* S141Δ*IPyAD*	*IPyAD* deletion, Δ*IPyAD::spm^r^*	This study
*B. velezensis* S141Δ*ipt*	*IPT* deletion, Δ*ipt::phle^r^*	This study
*B. velezensis* S141Δ*ipi*	*IPI* deletion, Δ*ipi::kan^r^*	This study

**Table 2:** Size of soybean nodules.

Size	Diameter of Nodule (mm)
Very large (VL)	more than 4
Large (L)	3–4
Medium (M)	2–3
Small (S)	Less than 2

## Supplementary Materials

Supplementary Materials are available online at https://www.mdpi.com/2076-2607/8/5/678/s1.
