# Supplementary material for: Co-Inoculation of Bacillus velezensis Strain S141 and Bradyrhizobium Strains Promotes Nodule Growth and Nitrogen Fixation
Source: Microorganisms. 2020 May 7;8(5):678. doi: 10.3390/microorganisms8050678 (PMC7284691; doi:10.3390/microorganisms8050678)
Supplement: Supplementary file 1 [file microorganisms-08-00678-s001.pdf]

microorganisms

Supplementary

Determinants derived from *Bacillus velezensis* strain S141 capable of increasing soybean production via *Bradyrhizobium* inoculation

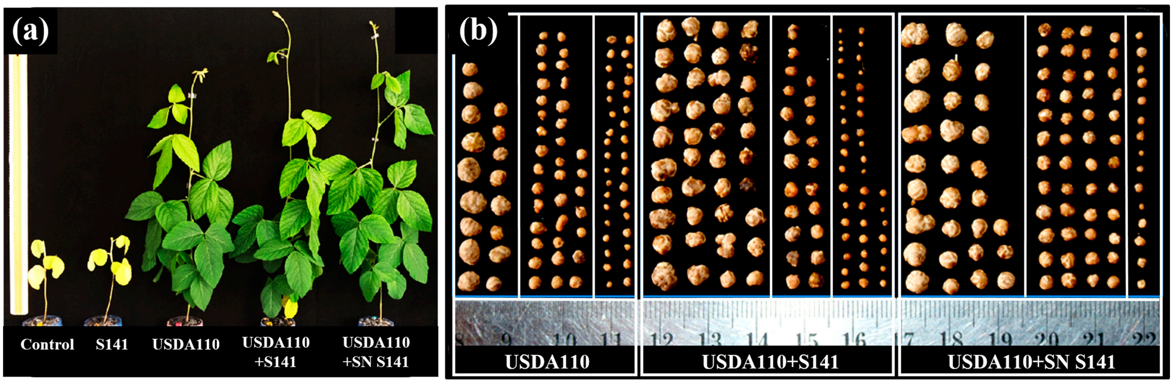

Figure S1. Soybean plant growth and the nodule size separation of soybean cultivar Chaing Mai 60 by co-inoculation with cells or supernatant of S141 and USDA110 at 45 DAI: (A) soybean growth under Leonard's jar experiments and (B) photograph of soybean nodules.

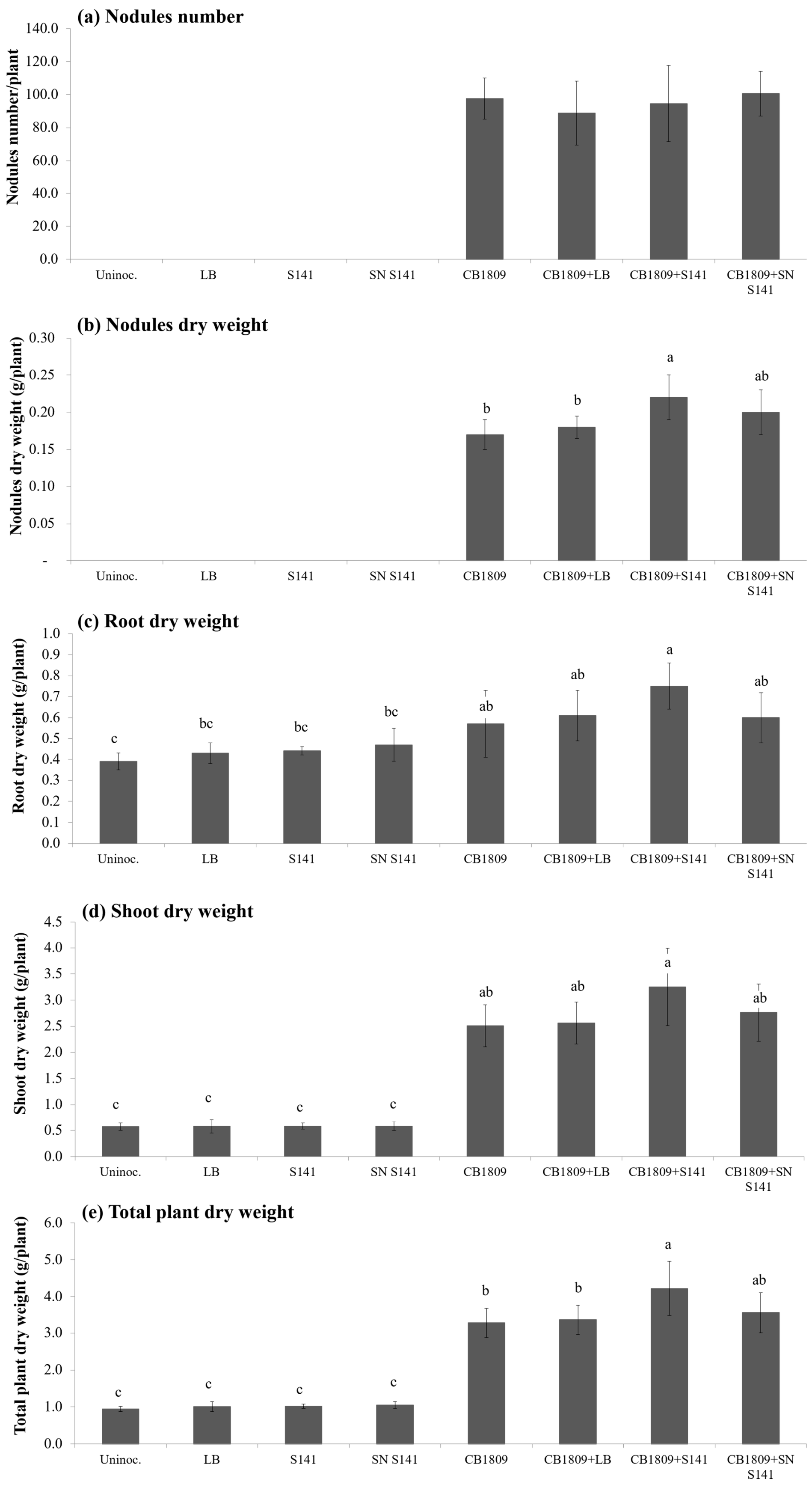

Figure S2. Plant growth parameters of soybean cultivar Chaing Mai 60 by co-inoculation with cells or supernatant of S141 and CB1809 at 45 DAI: (A) nodule numbers per plant (B) nodule dry weight,

(C) root dry weight, (D) shoot dry weight, and (E) total plant dry weight. Significance at *P* ≤*0.05* is indicated by mean standard error bars (n=8).

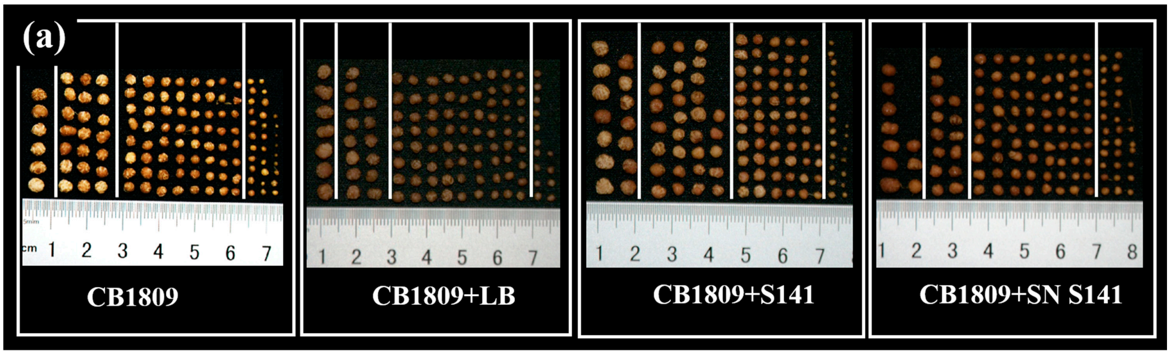

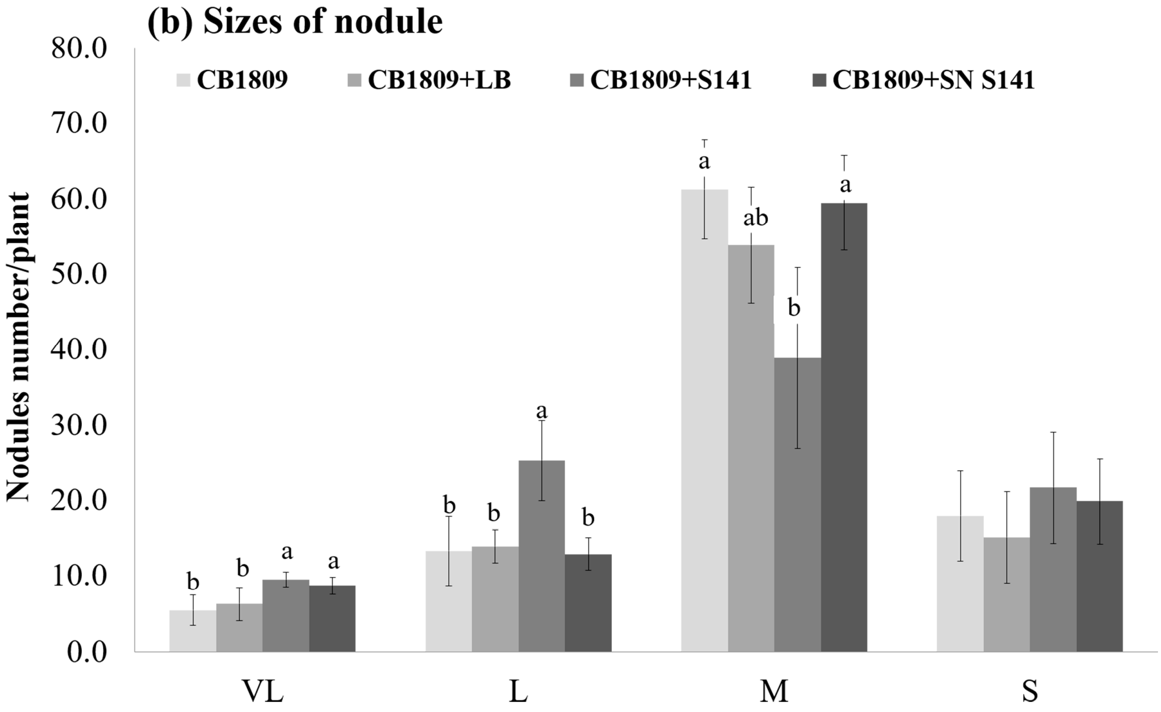

Figure S3. The nodule size separation of soybean cultivar Chaing Mai 60 by co-inoculation with cells or supernatant of S141 and CB1809 at 45 DAI: (A) photograph of soybean nodules and (B) nodule size separation of soybean. Significance at $p \leq 0.05$ is indicated by mean standard error bars ($n = 8$).

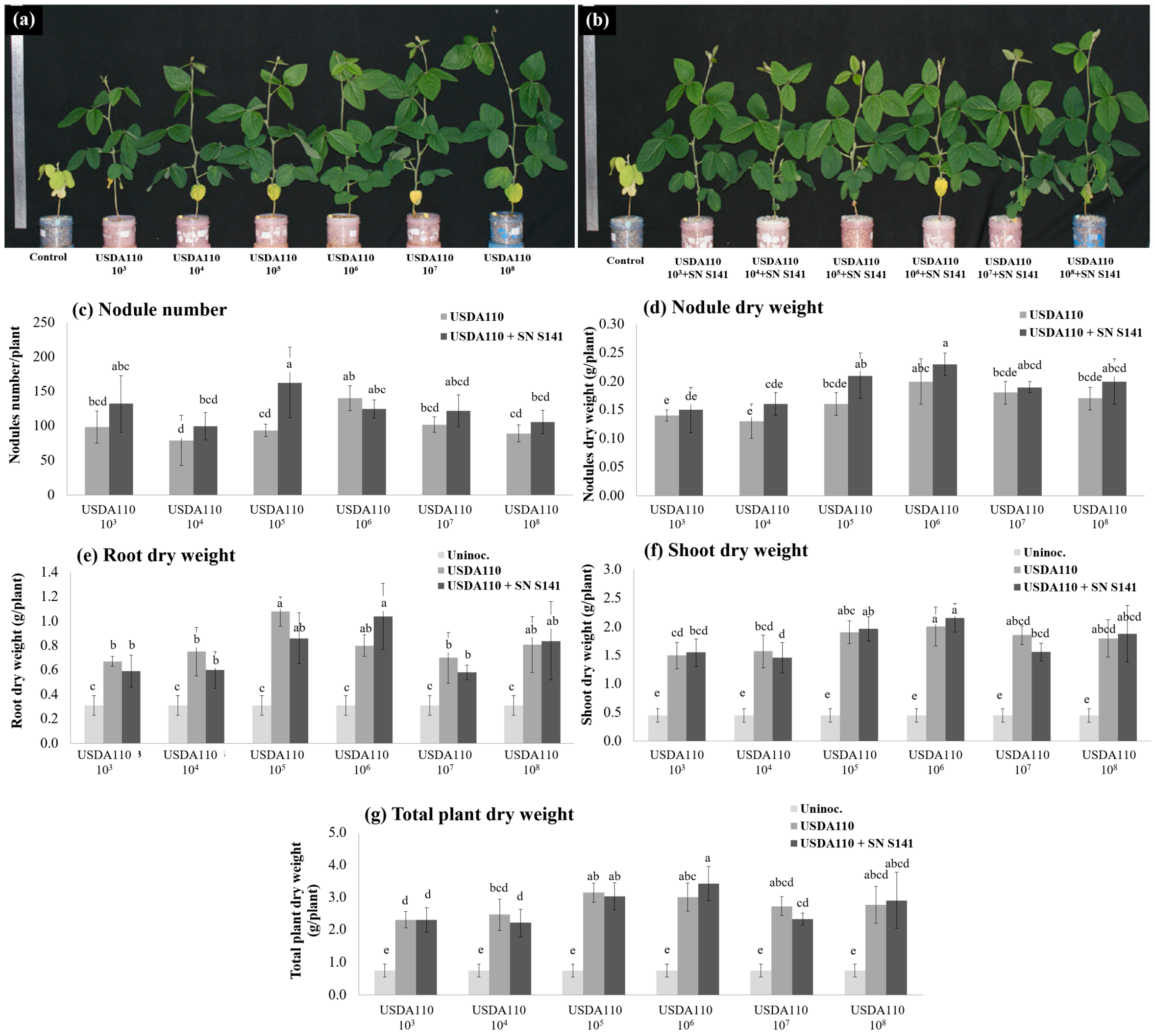

Figure S4. Effects of inoculation dose between supernatant of S141 with various doses of USDA110 on plant growth parameters of soybean cultivar Chaing Mai 60 at 45 DAI: (A) single inoculation, (B) co-inoculation between supernatant of S141 with various doses of USDA110 on soybean growth under Leonard's jar experiments, (C) nodule numbers per plant, (D) nodule dry weight, (E) root dry weight, (F) shoot dry weight, and (G) total plant dry weight. The numbers at the x-axis symbolize the varied inoculation doses of USDA110 are 10^3, 10^4, 10^5, 10^6, 10^7 and 10^8 CFU ml^{-1}. Significance at $p \leq 0.05$ is indicated by mean standard error bars (n = 5).

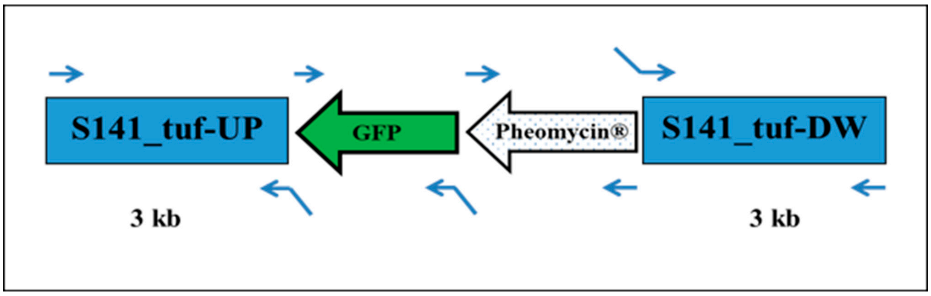

Figure S5. Construction of GFP and phleomycin resistance gene into S141 by insertion to *tuf* promoter gene.

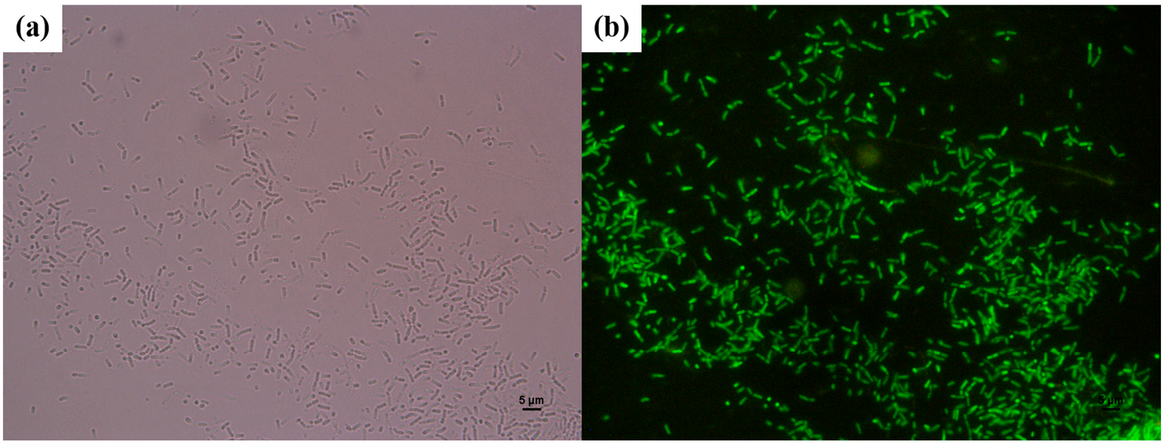

Figure S6. Visualization of green fluorescent protein (GFP) expression in S141: (A) bright-field and (B) fluorescence in free cells of S141.

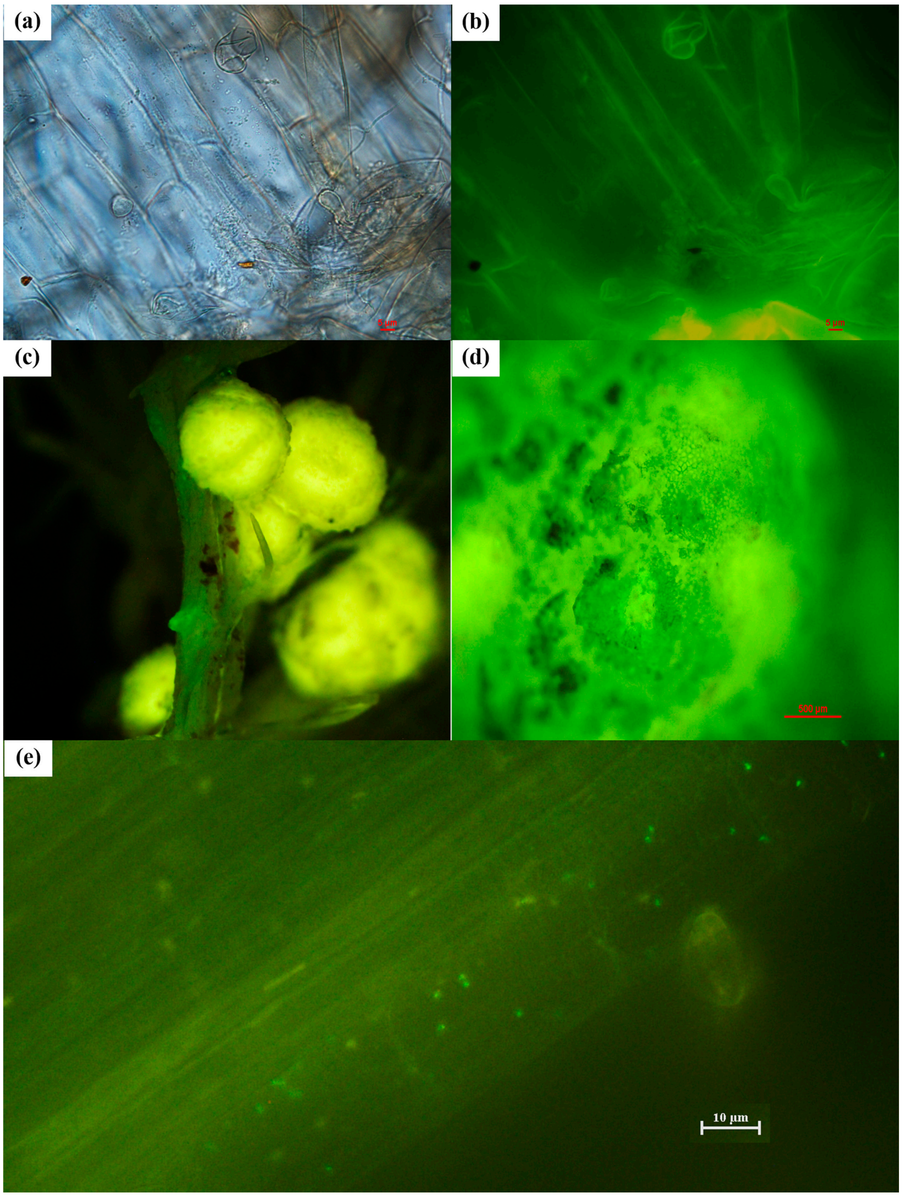

Figure S7. Colonization of S141 GFP-tagged strain on soybean rhizosphere: (A) bright-field and (B) fluorescence of S141 colonized around soybean root surface, (C) colonization of S141 around soybean

root surface, (D) colonization of S141 around soybean nodules, and (E) fluorescence of S141 colonized around soybean root surface.

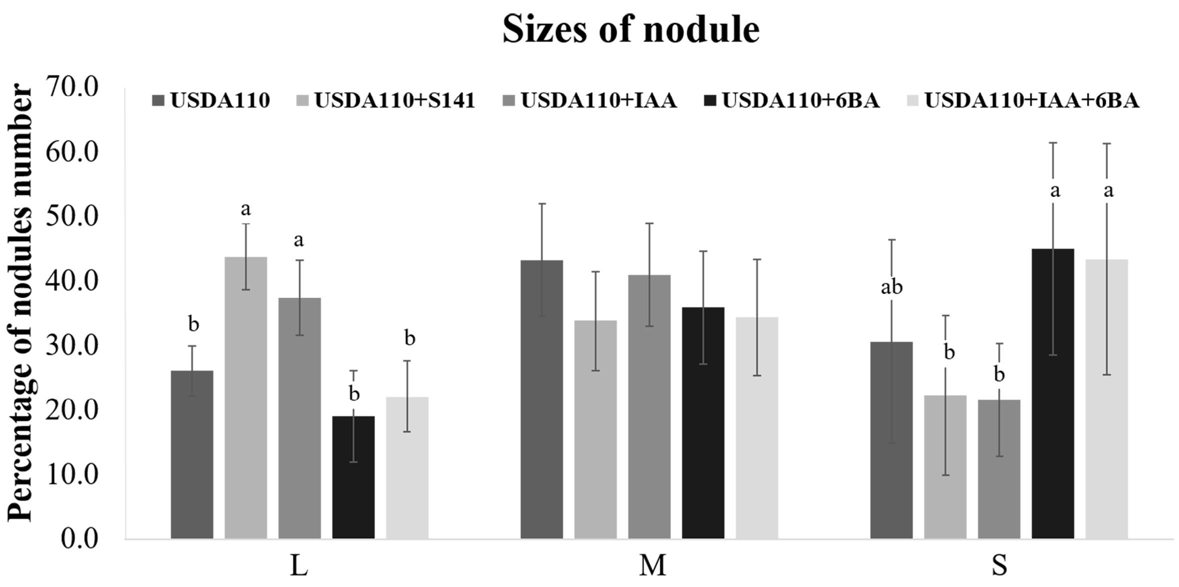

Figure S8. The percentage of nodule size separation of soybean cultivar Chaing Mai 60 by co-inoculation with S141 and USDA110 and/or supplemented with IAA and/or 6BA at 45 DAI. Significance at $p \leq 0.05$ is indicated by mean standard error bars (n = 8).

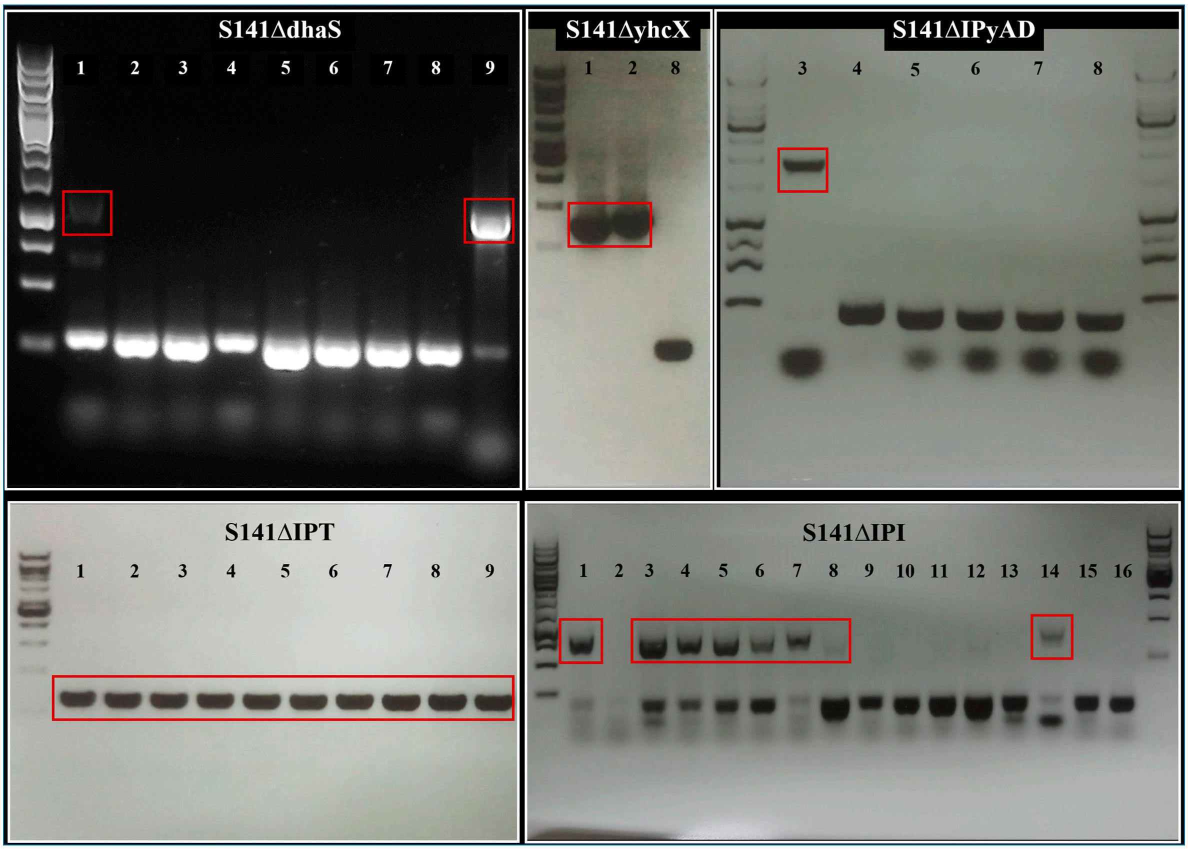

Figure 9. The verification of S141 mutant strains in *dhaS*, *yhcX* and *IPyAD* genes in auxin biosynthesis pathways and *IPT* and *IPI* genes in cytokinin biosynthesis pathways of S141. Red boxes indicate the DNA insertion bands to disruption of each gene.

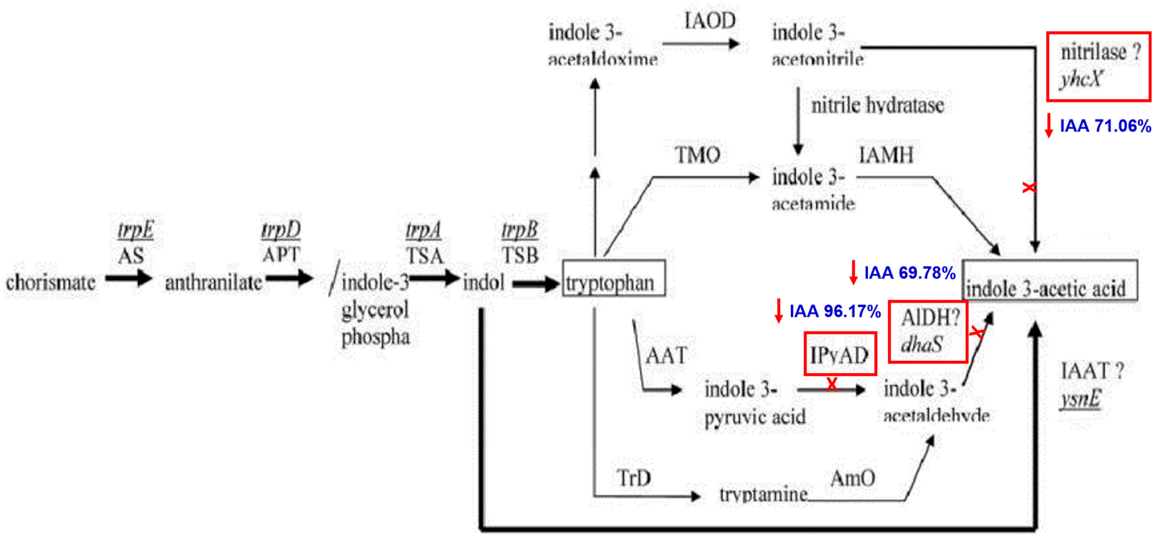

Figure S10. Disruption of *dhaS*, *yhcX* and *IPyAD* genes in tryptophan-dependent pathways of S141 indole-3-acetic acid (IAA) synthesis (Modified from Idris et al. (2007)). Red arrows indicate percentage of reduction of IAA production in S141 IAA related mutant strains.

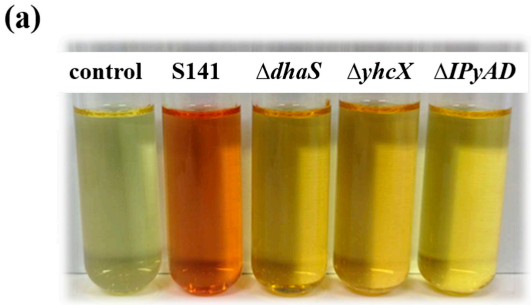

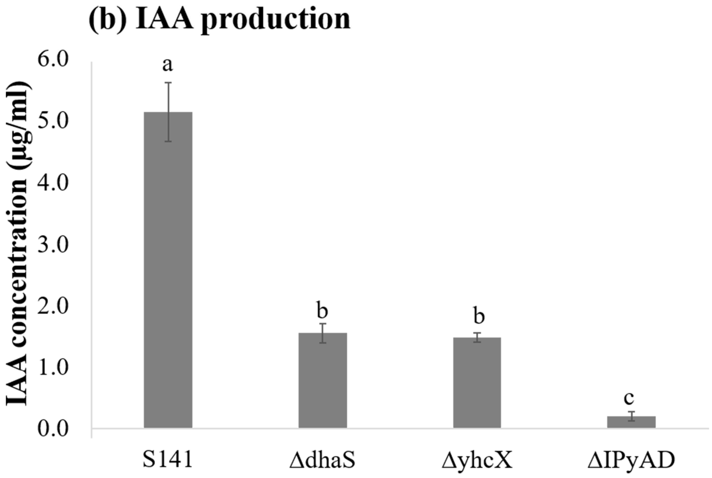

Figure S11. Quantification of the IAA production in S141 and S141 IAA related mutant strains (A) colorimetrically of IAA production and (B) IAA concentration in S141 and S141 IAA related mutant strains.

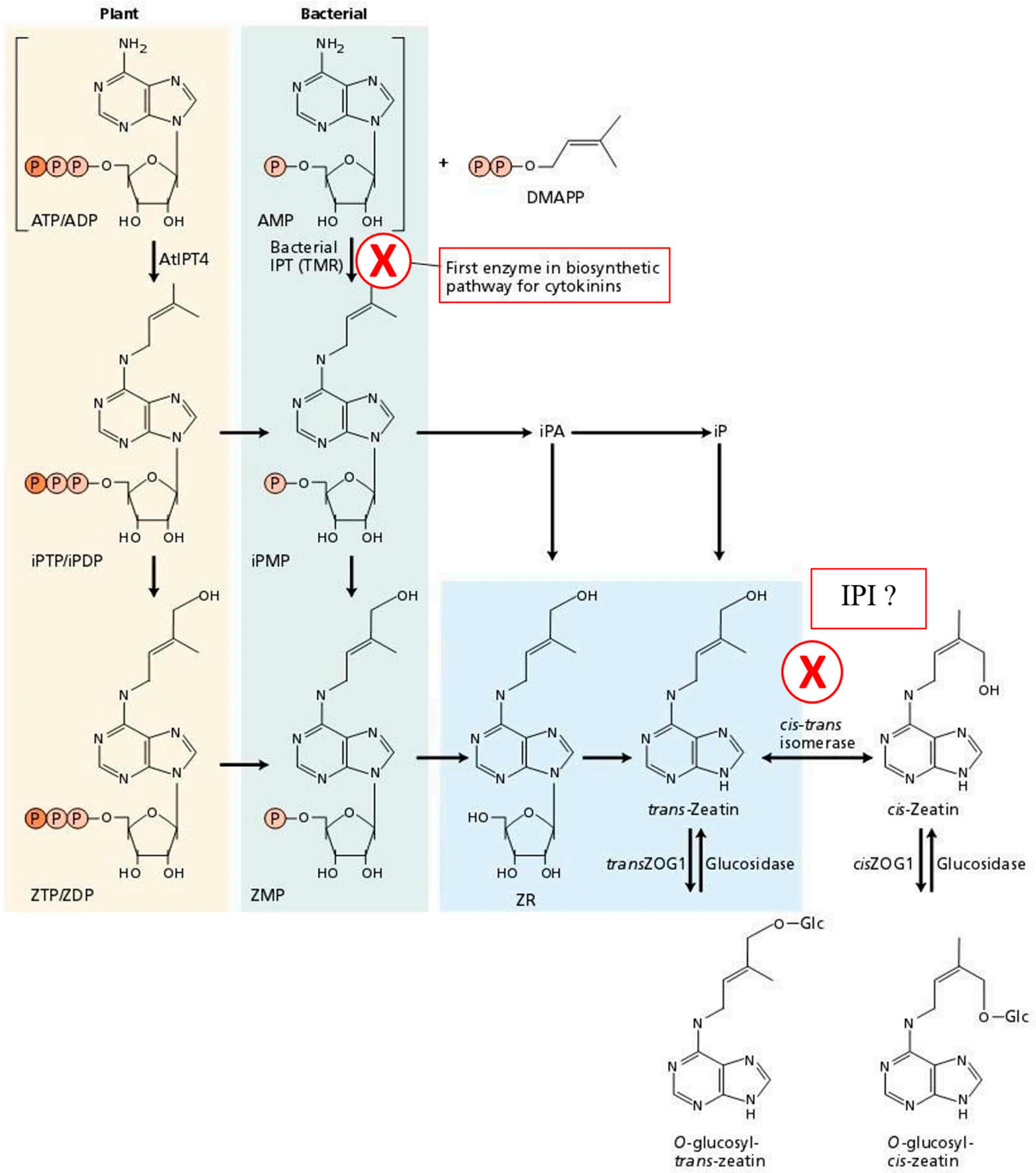

Figure S12. Disruption of *IPT* and *IPI* gene in cytokinin biosynthesis pathways of S141 (Modified from Kakimoto, (2003).

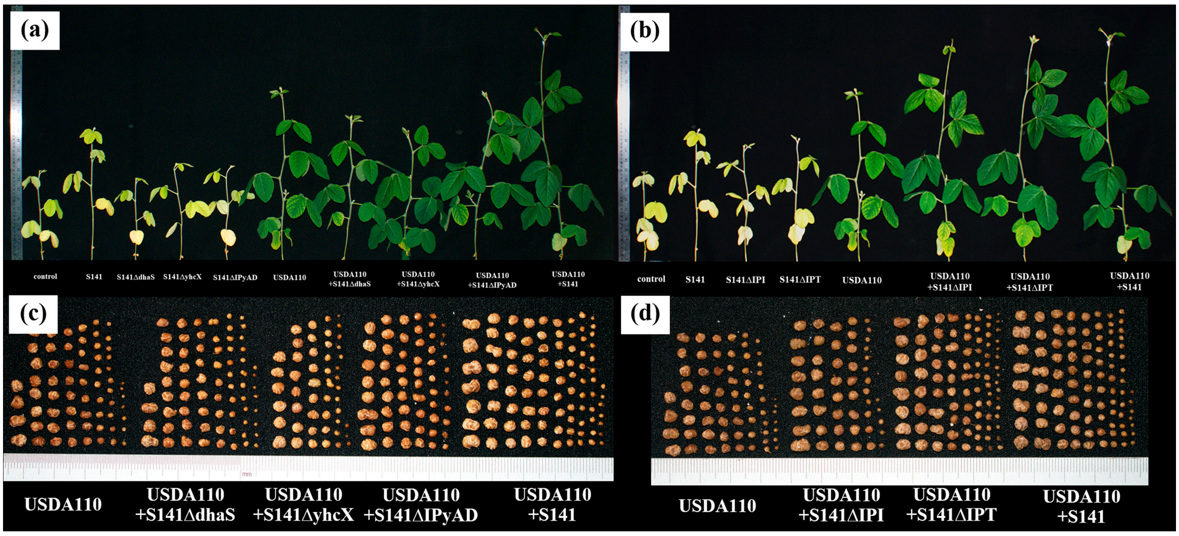

Figure S13. Soybean plant growth and the nodule size separation of soybean cultivar Chaing Mai 60 by co-inoculation with USDA110 and S141 or S141 mutant strains compared with single inoculation at 45 DAI: (A) and (B) soybean growth under Leonard's jar experiments and (C) and (D) photograph of soybean nodule.

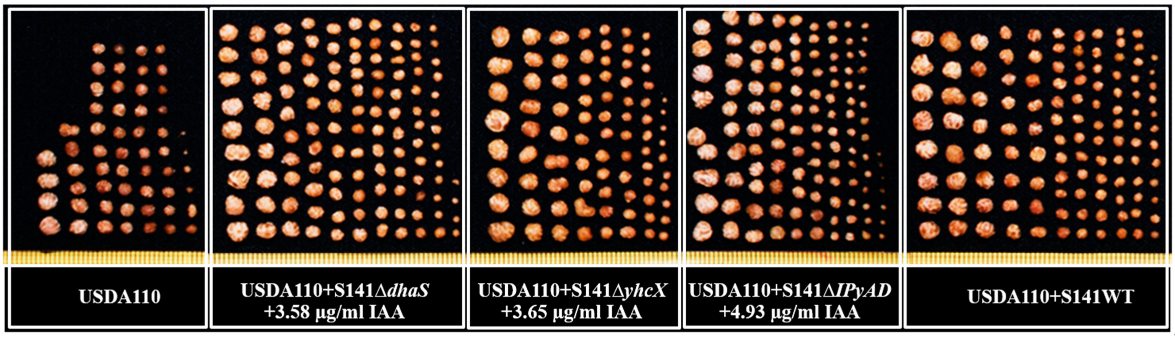

Figure S14. Photograph of soybean nodule cultivar Chaing Mai 60 by co-inoculation with USDA110 and S141 or S141 mutant strains supplemented with Indole-3-Acetic Acid compared with single inoculation at 45 DAI.

Table S1. Soil characteristics.

Soil characteristic	
Texture	sand
pH	7.064
EC (ms/cm)	0.238
Organic matter content (%)	0.722
Nitrogen (%)	0.036
Available P (ppm)	50.236
Exchangeable K (ppm)	69.00
Ca (ppm)	172.00

Table S2. Primers used in this study.

Primer	Sequences (5′→3′)	Annealing temperature used (°C)
tuf-uF	CGTTGTATTTGTCAACAAAATGGACAAAATC	60
tuf-uR	**GTACCCTCGACTCTAGATCACC**TTACTCAGTGATTGTAGAAACA	

	ACGCCTGAAC	
tuf-dF	**GCAATCGCCCTAATATATG**TGTTTCTACAATCACTGAGTAA	55
tuf-dR	GCGCTTAAGAACATCACGAGGATCTTTC	
gfp-F	GGTGATCTAGAGTCGAGGGTAC	55
gfp-R	**CCGAATAGCAAAAAACTGG**CGGGCTGCACTAGTGCTCG	
phl-F	CCAGTTTTTGCTATTCGG	55
phl-R	CATATATTAGGGCGATTGC	
dhaS_uF	TACATCCTGAGAAATGCAGATGGTC	58
dhaS_uF_nest	CGCGTAAATTTTTCTTCTCAGCATC	
dhaS_uR	**ATTTTAAGATACTGCACTATCAACACACTC**CCCACCTTGAAGATA CGGTGACAAAC	
dhaS_dF	**GAAAGTTACACGTTACTAAAGGGAATGTAG**GCGTCATTTAACA CGATCGTATGGT	58
dhaS_dR_nest	TAGGAAAACAGCTTGTCGATAAACG	
dhaS_dR	CCCTTTTAAAAGGCGTGTTTTTTC	
Erm_F	GAGTGTGTTGATAGTGCAGTATCTTAAAATTTTG	58
Erm_R	CTACATTCCCTTTAGTAACGTGTAACTTTCC	
yhcX_uF	ATGATAGCCTTTCACTTTGTAATGG	58
yhcX_uF_nest	TCAGCATATGACTGTCCTCAAATAA	
yhcX_uR	**TTTACTGGATGAATTGTTTTAGTACCTAGA**TTTCATTTCATATTG AATGACGCAG	
yhcX_dF	**CCAATTCACTGTTCCTTGCATTCTAAAACC**AAGATTTACTCCTTC GAGGAATTTGC	58
yhcX_dR_nest	ATAGTTCGGTGTCTGAAGTCGTCAC	
yhcX_dR	TAAGCGGTGTCAATCTTTTTTTGTC	
Kan_F	TCTAGGTACTAAAACAATTCATCCAG	58
Kan_R	GGTTTTAGAATGCAAGGAACAGTGAATTGG	
IPyAD_uF	ATTAAGAGATGAATCGGCTTGATGC	58
IPyAD_uF_nest	CTCTTTCTCCTATGCCGTTAATGGC	
IPyAD_uR	**CTGTTCAATAAAGCTGACCGTTAGCGTTTA**GGAAAAATTATTG AAACGCAAATTCC	
IPyAD_dF	**ACGCTTTATTACTTTAATTTAGTGAAGCTT**GATTTCAGCAGGAT CAATATCGATATG	58
IPyAD_dR_nest	TAATATGCCATTGTTGGATACACGG	
IPyAD_dR	AACGAGTGGAAAGATATCACACAGAAC	Cont.

Table S2. Primers used in this study (continued).

Primer	Sequences (5′→3′)	Annealing temperature used (°C)
Spm_F	TAAACGCTAACGGTCAGCTTTATTG	58
Spm_R	AAGCTTCACTAAATTAAAGTAATAAAGCGTTCTC	
IPT_uF	CCCTTGGCAAAACATCACTT	55
IPT_uF_nest	TGATGAAATGCTGCTTGGAG	
IPT_uR	**CCGAATAGCAAAAAACTGG**GATTTCCGCATTCAGCATTT	
IPT_dF	**GCAATCGCCCTAATATATG**CAGGCGATCGGCTATAAAGA	55
IPT_dR_nest	AGCCGATTTCGTCTTCTTCA	
IPT_dR	AACCAAGGTTGCAGGAAATG	
Phl_F	CCAGTTTTTGCTATTCGG	55
Phl_R	CATATATTAGGGCGATTGC	
IPI_uF	ACTTGATGAAGCGCCTCTGT	55
IPI_uF_nest	TCAACAACCATCCCCATCTT	
IPI_uR	**CTGGATGAATTGTTTTAGTACCTAGA**CAAAAGACGCCTTTGGTCAT	
IPI_dF	**CCAATTCACTGTTCCTTGCATTCTAAAACC**TAAAAATCGGCGAACTGC	55

	TT	
IPI_dR_nest	TCCGGCAGTTCATGATACAA	
IPI_dR	GGAAATGAAGCGGATTCAAA	
Kan_F	TCTAGGTACTAAAACAATTCATCCAG	58
Kan_R	GGTTTTAGAATGCAAGGAACAGTGAATTGG	

Table S3. Comparison of genes involved in indole-3-acetic acid (IAA) production between *B. velezensis* FZB42 with S141.

**Gene in *Bacillus velezensis* S141**		**Gene in *Bacillus velezensis* FZB42 (Reference strain)**	
**Gene name**	**Gene description**	**Gene name**	**Gene description**
menF	Isochorismate synthase	*trpE*	Anthranilate synthase
dhbC	Isochorismate synthase	*trpE*	Anthranilate synthase
trpA	tryptophan synthase (alpha subunit)	*trpA*	Tryptophan synthase (alpha subunit)
trpB	tryptophan synthase (beta subunit)	*trpB*	Tryptophan synthase (beta subunit)
trpF	phosphoribosylanthranilate isomerase	*trpF*	Phosphoribosyl anthranilate isomerase
trpC	indol-3-glycerol phosphate synthase	*trpC*	Indol-3-glycerol phosphate synthase
trpD	Anthranilate phosphoribosyltransferase	*trpD*	Anthranilate phosphoribosyl transferase
trpE	Anthranilate synthase	*trpE*	Anthranilate synthase
pabB	para-aminobenzoate synthase subunit A	*trpE*	anthranilate synthase
NADP-dependent glyceroldehyde 3-phosphate dehydrogenase	NADP-dependent glyceroldehyde 3-phosphate dehydrogenase	*dhaS*	NADP-dependent indole-3-aldehyde dehydrogenase
aldehyde dehydrogenase	NAD-dependent aldehyde dehydrogenase	*dhaS*	NADP-dependent indole-3-aldehyde dehydrogenase
gabD	NAD-dependent aldehyde dehydrogenase	*dhaS*	NADP-dependent indole-3-aldehyde dehydrogenase
gbsA	NAD-dependent aldehyde dehydrogenase	*dhaS*	Indole-3-acetaldehyde dehydrogenase
aldehyde dehydrogenase	NAD-dependent aldehyde dehydrogenase	*dhaS*	NADP-dependent indole-3-aldehyde dehydrogenase
1-pyroline-5-carboxylate dehydrogenase	NAD-dependent aldehyde dehydrogenase	*dhaS*	NADP-dependent indole-3-aldehyde dehydrogenase
methylmalonate-semialdehyde dehydrogenase	NAD-dependent aldehyde dehydrogenase	*dhaS*	NADP-dependent indole-3-aldehyde dehydrogenase

cont.

Table S3. Comparison of genes involved in indole-3-acetic acid (IAA) production between *B. velezensis* FZB42 with S141 (continued).

**Gene in *Bacillus velezensis* S141**		**Gene in *Bacillus velezensis* FZB42 (Reference strain)**	
**Gene name**	**Gene description**	**Gene name**	**Gene description**
AldX	NAD-dependent aldehyde dehydrogenase	*dhaS*	NADP-dependent indole-3-aldehyde dehydrogenase
ywdH	Aldehyde dehydrogenase	*dhaS*	NADP-dependent indole-3-aldehyde

			dehydrogenase
1-pyroline-5-carboxylate dehydrogenase	1-pyroline-5-carboxylate dehydrogenase	*dhaS*	NADP-dependent indole-3-aldehyde dehydrogenase
acetyltransferase	Acetyltransferase	*ysnE*	IAA transacetylase
amidohydrolase	Amidohydrolase	*yhcX*	Nitrilase, hydrolase carbon-nitrogen family
yhcX	Carbon-nitrogen hydrolase protein family (amidohydrolase)	*yhcX*	Nitrilase, hydrolase carbon-nitrogen family
trpP	Tryptophan transporter		
hypothetical protein	Anthranilate/para-aminobenzoate		
pabA	para-aminobenzoate/anthranilate synthase glutamate transferase component II		
Translocator protein peripheral-receptor	Tryptophan-rich sensory protein		
